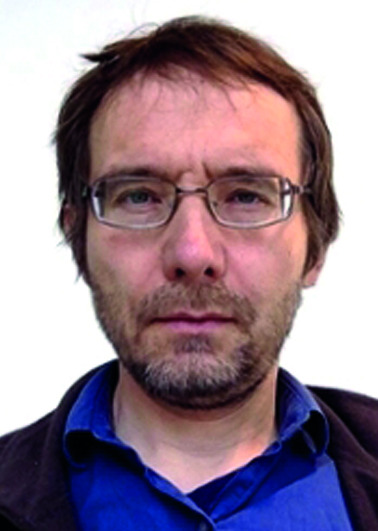# Mono‐ADP‐Ribosylation of Peptides: An Overview of Synthetic and Chemoenzymatic Methodologies

**DOI:** 10.1002/cbic.202400440

**Published:** 2024-09-05

**Authors:** Hugo Minnee, Jeroen D. C. Codée, Dmitri V. Filippov

**Affiliations:** ^1^ Bio-Organic Synthesis Leiden Institute of Chemistry Leiden University Leiden 2300 RA Netherlands

**Keywords:** mono-ADP-ribosylated peptides, solid-phase peptide synthesis, chemoenzymatic synthesis, pyrophosphate, glycosylation

## Abstract

Adenosine diphosphate (ADP)‐ribosylation is a ubiquitous post‐translational modification that regulates vital biological processes like histone reorganization and DNA‐damage repair through the modification of various amino acid residues. Due to advances in mass‐spectrometry, the collection of long‐known ADP‐ribose (ADPr) acceptor sites, e. g. arginine, cysteine and glutamic acid, has been expanded with serine, tyrosine and histidine, among others. Well‐defined ADPr‐peptides are valuable tools for investigating the exact structures, mechanisms of action and interaction partners of the different flavors of this modification. This review provides a comprehensive overview of synthetic and chemoenzymatic methodologies that enabled the construction of peptides mono‐ADP‐ribosylated on various amino acids, and close mimetics thereof.

## Graphical abstract

Adenosine diphosphate (ADP)‐ribosylation is a post‐translational modification that regulates key biological processes. It occurs on a wide range of amino acids, including arginine, cysteine, serine, and glutamic acid. Well‐defined ADP‐ribosylated peptides are valuable tools for investigating the structures, mechanisms of action, and interaction partners of this modification. This article provides a comprehensive overview of chemical and chemoenzymatic methodologies for the construction of mono‐ADP‐ribosylated peptides.

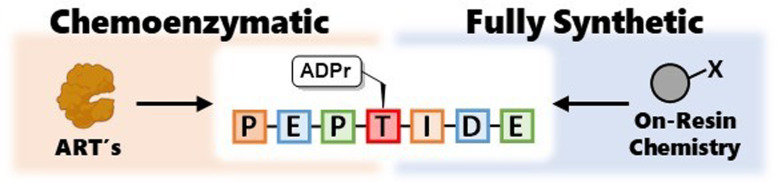

## 1 Introduction

**Figure 1:**
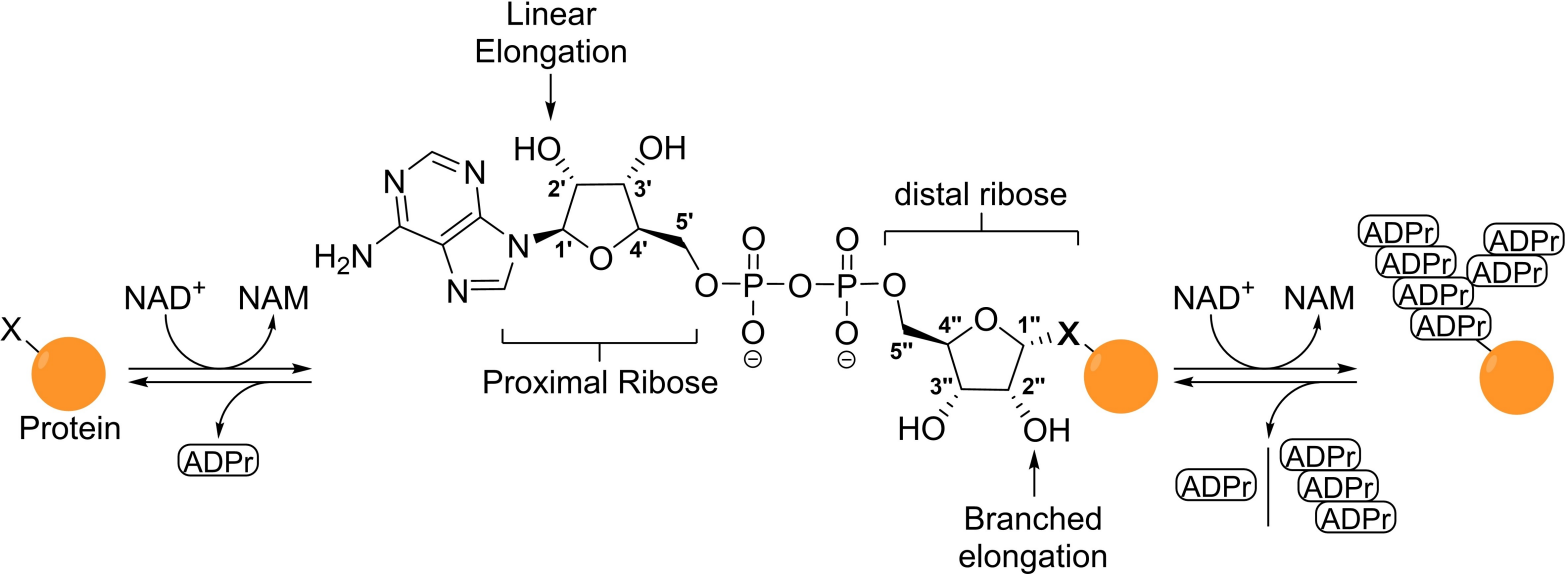
Schematic overview of ADP‐ribosylation where NAD^+^ is consumed and the ADPr moiety is covalently attached to a defined nucleophilic side chain (X=O, N or S) in an α‐selective manner. Numbering denotation and nomenclature used to discriminate between the two ribofuranosides is included. ADPr=Adenosine diphosphate ribose, NAD=Nicotinamide adenine dinucleotide and NAM=nicotinamide.

Nicotinamide adenine dinucleotide (NAD^+^) is well known for its role as a redox co‐factor in various metabolic biosynthesis pathways as well as in energy production through processes like Krebs cycle and oxidative phosphorylation. However, it actually also serves as an essential substrate in many biochemical processes.[^1^, ^2^, ^3^] It is not widely known, but NAD^+^ is used as a reagent in a common post‐translational modification (PTM) called adenosine diphosphate (ADP)‐ribosylation. In this process, one or more ADP‐ribose (ADPr) molecules are covalently transferred to a nucleophilic amino acid side chain of the target protein (see Figure 1). ADP‐ribosylation of proteins is essential for a multitude of biological processes and a subject of active research.[^4^, ^5^, ^6^, ^7^] In this review, we briefly discuss the biochemistry of ADP‐ribosylation, highlighting the transferase and hydrolase enzymes involved in the synthesis and break‐down of ADPr chains and offer an in‐depth overview of the chemoenzymatic and synthetic methodologies available for the construction of well‐defined mono‐ADP‐ribosylated (MARylated) peptides which are useful molecular tools for studying the biology of ADPr.[^8^, ^9^, ^10^]

## 2 ADP‐Ribosylation

The initial isolation of an acid‐insoluble adenylic polymer from nuclear extracts from hen liver cells by Chambon and coworkers^[11]^ over five decades ago and its subsequent identification as ADP‐ribosyl polymer^[12]^ opened up the field of ADP‐ribosylation. The exact structure of the poly‐ADPr (PAR) chain was elucidated by Miwa *et al*.[^13^, ^14^, ^15^] in several spectroscopic studies on fragments that were obtained after the digestion of poly‐ADPr with snake venom phosphodiesterase. Shortly after, the hypothesis of a purely linear chain was challenged when a branching fragment was isolated.^[14]^ The frequency of branching in the ADPr‐chains could be estimated to be only 0.7–0.8 % with the use of a high‐performance liquid‐chromatography‐based fluorescent assay (HPLC) assay,^[16]^ which also explains why this phenomenon remained unnoticed in previous investigations. Significant insight has been gained over time regarding the structure and function of poly‐ADP‐ribose, especially on the regulatory role of PARP1 in the DNA damage response.[^17^, ^18^] However, mono‐ADP‐ribosylation (MARylation) is much less well understood with respect to its molecular targets, involved enzymes and, most importantly, the biological relevance of this modification.[^19^, ^20^] During this modification a single ADPr‐unit is attached to a specific nucleophilic sidechain of the target protein (Figure 1). The newly established bond between the protein and the so‐called anomeric centre (1’’) of the distal ribose is known as a glycosidic bond and can have two distinct stereochemical orientations. The glycosidic bond is referred to as “α” when the linkage is situated at the same side of the furanose ring as the 4’’‐linked phosphate, while it is assigned “β” when it is oriented in the opposite direction.

### 2.1 (ADP‐Ribosyl)‐Transferases

**Figure 2:**
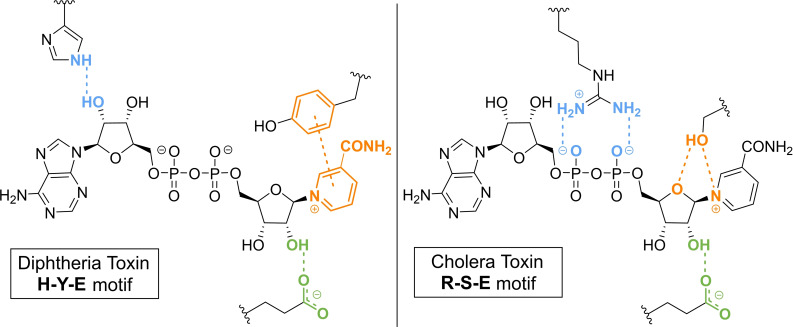
The catalytic triad of diphtheria toxin (left) and cholera toxin (right), which are both highly conserved among ADP‐ribosylating bacterial toxins, including their essential interactions with the NAD^+^ substrate.

Pathogenic bacterial exotoxins were among the first to be identified as enzymes with (ADP‐ribosyl)transferase activity, introducing this modification to yield a unique pathology.^[21]^ For instance, a multidomain protein called diphtheria toxin (DT) secreted by *Corynebacterium diphtheriae* effectively inhibits host protein biosynthesis by modifying the essential elongation factor 2 (EF2) with a single ADP‐ribose moiety.^[22]^ By means of tryptic digestion, the exact modification site on EF2 was found to be a unique residue termed ‘diphthamide’[^23^, ^24^] that is derived from histidine through a multi‐step biosynthesis pathway.^[25]^ NMR analysis of the ADP‐ribosylated fragment, conducted by Oppenheimer and Bodley, indicated that, similar to the linear and branched elongation of PAR chains, the modification is exclusively α‐selective with respect to the configuration of the glycosidic bond formed.^[26]^ Similarly, cholera toxin (CT)[^27^, ^28^] and pertussis toxin (PT),^[29]^ excreted by *Vibrio cholerae* and *Bordetella pertussis*, respectively, inhibit G‐proteins by mono‐ADP‐ribosylation (MARylation) of the target's α‐subunit and, as a consequence, interfere with cyclic adenosine monophosphate (cAMP) mediated signaling pathways. Despite the similarities in the target proteins, CT and PT have distinct amino acid substrate specificity as the former was observed to modify arginine residues in an α‐selective manner (even though racemization occurs after the introduction),^[30]^ while the latter constructs α‐ribosyl linkages to cysteine.^[31]^ With the aid of both crystallographic and mutagenic data, essential amino acids in the catalytic domain of (ADP‐ribosylating) bacterial toxins could be identified.[^32^, ^33^] In the case of DT, an imidazole residue of histidine was observed to interact with the proximal ribose of NAD^+^ while a tyrosine moiety engages in π–π stacking with the aromatic system of nicotinamide (Figure 2). Finally, the glutamic acid carboxylate group correctly positions the distal ribose for an incoming nucleophilic attack through hydrogen bonding.[^34^, ^35^, ^36^, ^37^] Although glutamic acid was found to fulfil a similar role in CT and DT, NAD^+^ binding in CT is assisted by arginine and serine instead of the histidine and tyrosine residues observed for DT. The guanidine group of arginine establishes electrostatic interactions with the negatively charged pyrophosphate and serine arranges hydrogen bonds with the distal ribose and nicotinamide.[^34^, ^37^, ^38^] Sequence alignment and three‐dimensional structural studies of the bacterial (ADP‐ribosyl)transferases not only disclosed that the active sites are superimposable but also revealed that the above‐described catalytic triads (H‐Y‐E and R‐S‐E) are highly conserved throughout the family.[^39^, ^40^]

Parallel to the increasing understanding of toxin‐mediated ADP‐ribosylation, advances were made in the search for the eukaryotic orthologs. Gill *et al*. performed *in vitro* studies with calf thymus extracts and demonstrated that poly‐ADP‐ribosylation (PARylation) activity was stimulated by DNA, especially when carrying double‐stranded breaks.[^41^, ^42^] These findings undeniably emphasized that ADP‐ribosylation is not only an important bacterial virulence trait, but is also an integral part of the normal functioning and regulation of cell physiology in mammals. A breakthrough occurred several years later when the gene encoding the protein responsible for PAR production termed PARP1 (formerly referred to as PARP or poly(ADP‐ribosyl)transferase) was finally isolated, sequenced and cloned by different labs.[^43^, ^44^, ^45^] For a long time, it was believed that PARP1 was the only enzyme mediating ADP‐ribosylation in mammalian cells until comparative analysis of the public nucleotide and protein sequence databases^[46]^ revealed the identity of 20 additional members of the (ADP‐ribosyl)transferase family in humans.[^47^, ^48^, ^49^, ^50^, ^51^, ^52^, ^53^, ^54^, ^55^, ^56^, ^57^] Moreover, the earlier recognized catalytic motifs of diphtheria (H‐Y‐E) and cholera (R‐S‐E) toxins correlate closely with the eukaryotic orthologs, which are distributed accordingly into two subgroups: DT‐like and CT‐like (ADP‐ribosyl)transferases (ARTDs and ARTCs).^[58]^ The ARTD family includes all intracellular enzymes, some of which are nuclear‐localized. It is the most abundant family of ADPr‐transferases, comprising a total of seventeen members (PARP1‐4, tankyrase 1 and 2, and PARP6‐16). In contrast, the enzymes belonging to the ARTC clade are either expressed at the cell surface or secreted in extracellular components (ARTC1 and 3–5).[^56^, ^59^]

Since the early discovery of diphthamide,^[23]^ arginine,^[60]^ cysteine^[31]^ and asparagine^[61]^ as acceptor sites for ADP‐ribosylating bacterial toxins, other endogenous modification sites have been identified *in vitro* using radioactive substrates in combination with purified or recombinantly expressed enzymes.[^62^, ^63^, ^64^, ^65^] However, the true scope of ADP‐ribosylation sites and the prevalence of the modification was not recognized until advances in mass‐spectrometry (MS) enabled the proteome‐wide analysis of the cellular ADP‐ribosylation.[^66^, ^67^, ^68^, ^69^, ^70^, ^71^, ^72^, ^73^] Serine has recently emerged as the primary target for ADP‐ribosylation, especially in DNA damage response,^[68]^ but residues from the early ADP‐ribosylation studies, such as glutamate,^[66]^ aspartate,^[66]^ arginine,[^71^, ^72^] lysine[^62^, ^67^, ^69^] and cysteine,^[70]^ and newer additions like threonine,^[70]^ tyrosine[^71^, ^74^] and histidine[^70^, ^73^] were found to be frequently modified as well. Covering nearly half of all amino acid residues, it comes as little surprise that ADP‐ribosylation is involved in the regulation of numerous vital cellular processes such as chromatin maintenance,^[75]^ DNA‐damage repair,^[76]^ protein degradation,^[77]^ cytosolic RNA processing,^[78]^ apoptosis^[79]^ and immune response[^80^, ^81^] and that abnormal functioning of the involved enzymes has been linked to cancer,^[82]^ metabolic diseases^[83]^ and neurological disorders.^[84]^

Nevertheless, the underlying mechanisms dictating substrate specificity of ADPr‐transferases are still poorly understood due to the complex regulation that seems to adjust the target preferences at various levels. For example, the selectivity for arginine residues observed for the catalytically active ARTC1‐2 members is typical for cholera toxin‐like (R‐S‐E triad) transferases,^[56]^ while PARPs have a clear mismatch with their diphtheria toxin‐like ancestor and predominantly target acidic residues (glutamate and aspartate)^[85]^ instead of diphthamide and analogues thereof. Furthermore, the canonical activity of PARP1 was found to be redirected to glutamate and aspartate residues on histone H2B^[86]^ through interaction with the chaperone NMNAT1 or to serine residues of various substrates when complexed with the co‐factor histone parylation factor (HPF1).^[5]^ The steric and charge effects of phosphorylated residues have been shown to affect the ADP‐ribosylation of surrounding residues, and *vice versa*. This demonstrates the additional effect of cross‐talk with other PTMs modifying the target specificity of the corresponding “writer” enzymes.[^70^, ^87^] It is noteworthy that PARylation activity has so far only been found in a limited number of human (ADP‐ribosyl)transferases. PARP2^[49]^ and tankyrase 1–2[^47^, ^55^, ^88^] were demonstrated, in addition to the founding member PARP1,^[89]^ to extend the MARylated modification with additional ADPr units. Most members of the PARP family thus MARylate rather than PARylate the target proteins. It is noteworthy that despite MARylation being apparently more common than PARylation, the latter process has received more attention from the scientific community. We believe that the biology of MARylated proteins is complex and diverse and, more importantly, that synthetic mono‐ADP‐ribosylated peptides can be instrumental in uncovering the yet undiscovered biology of MARylated proteins.

### 2.2 ADPr‐Glycohydrolases

The reversibility of ADP‐ribosylation has been known since the early demonstrations of PAR degradation by partially purified rat liver and calf thymus extracts containing an enzyme termed PARG.[^90^, ^91^] Interestingly, PARG was shown to specifically catalyze the hydrolysis of ribose‐ribose bonds, while leaving the protein‐ribose linkage intact.[^91^, ^92^] Although PARG was actually found to revert ester‐linked mono‐ADPr modifications very recently,^[93]^ the original observation implied the existence of complementary hydrolases that revert the protein‐linked ADPr moiety. The PARG gene was already cloned in the late 90s,^[94]^ but no structural data was available until the first crystal structure was solved by Ahel *et al*.^[92]^ This study revealed that the hydrolysis mechanism likely proceeds via a reactive oxocarbenium intermediate upon activation of the *O*‐glycosidic linkage through interactions with a nearby glutamate residue, and it also explained that PARG predominantly acts as an exo‐acting enzyme because the presence of a substituent on the 2’‐OH of the proximal ribose would require major reorganizations of the active site. Furthermore, a comparative analysis of the PARG catalytic domain with available protein structures indicated that its ADPr‐binding fold is shared with macrodomain proteins, which suggests that PARG is a distant member of this widespread family. Although many macrodomains function as ADPr‐binders,[^95^, ^96^] some members from the macrodomain type (MacroD1 and ‐D2) and ALC1‐like (C6orf130, better known as TARG1) subclasses possess catalytic activity and were found to function as *O*‐acetyl‐ADPr (OAADPr) deacetylases.[^97^, ^98^] The chemical similarities between OAADPr and the glycosidic ester linkage of glutamate‐ADPr led to the assumption that these three macrodomain proteins potentially function as (ADP‐ribosyl)hydrolase for acidic residues. Two separate laboratories confirmed this hypothesis and showed that all three candidates liberated ADPr in a biochemical screening with MARylated peptides that were generated by PARP10.[^99^, ^100^]

Independent of the developments described above, another ADPr‐glycohydrolase with an exclusive specificity towards arginine residues was isolated from turkey erythrocytes and subsequently characterized as the founding member ARH1 of the evolutionary distinct ADPr‐hydrolase family.[^101^, ^102^] Several years later, the human homolog was cloned^[103]^ and two additional members of the human ARH family, designated ARH2 and ARH3, were discovered in a sequence alignment study.^[56]^ Apart from its localization in heart tissue, little is known about ARH2.^[104]^ It was demonstrated that ARH3 is capable of processing linear PAR chains and that it is unable to degrade PAR branching points^[105]^ or remove mono‐ADPr from cysteine, diphthamide and arginine.^[106]^ Indeed, the latter substrate was found to function as an inhibitor with nanomolar affinity.^[107]^ Nevertheless, the known substrate scope of ARH3 has expanded ever since and now covers α‐NAD^+^,^[108]^ OAADPr,^[109]^ ADPr‐5’P DNA^[110]^ and most importantly, serine‐linked mono‐ADPr.^[111]^ At first sight, the active sites of ARH1 and ARH3 appear structurally very similar, which is surprising given their distinct substrate preferences, and they both contain a binuclear magnesium center required for catalytic activity.^[112]^ Closer inspection of the active site residues made it clear that substrate specificity is tightly regulated by seemingly subtle differences in the Mg(II)‐coordination spheres, which have been described in detail by Rack *et al*.[^105^, ^112^]

## 3 Chemical and Enzymatic Strategies for the Synthesis of Mono‐ADP‐Ribosylated Peptides

The understanding of the mechanisms of protein ADP‐ribosylation has been greatly assisted by the production of well‐defined MARylated oligopeptides and analogues thereof. These molecular tools have enabled the production and evaluation of recombinant ADP‐ribose specific antibodies,^[113]^ provided valuable structural insights of the modification,[^9^, ^30^, ^114^] assisted in the elucidation of the substrate specificity of ADPr‐glycohydrolases,[^9^, ^115^, ^116^] and were shown to be of use as probes for pull‐down experiments to aid in the identification of novel ADPr‐binders.[^8^, ^117^] The synthetically prepared MARylated peptides hold potential as the internal standards for HPLC and MS‐based analyses. Here, we present an overview of the reported methodologies towards the synthesis of MARylated peptides and proteins. This review discusses methods for synthesizing mono‐ADPr‐peptides with a native or artificial linkage between the distal ribose of ADPr and the protein/peptides. For the purpose of our discussion the methods towards native MARylated peptides and proteins are divided into chemoenzymatic and fully synthetic strategies.

### 3.1 Enzymatic Construction of Native ADP‐Ribose Linkages

#### 3.1.1 Arginine and Cysteine

**Figure 3:**
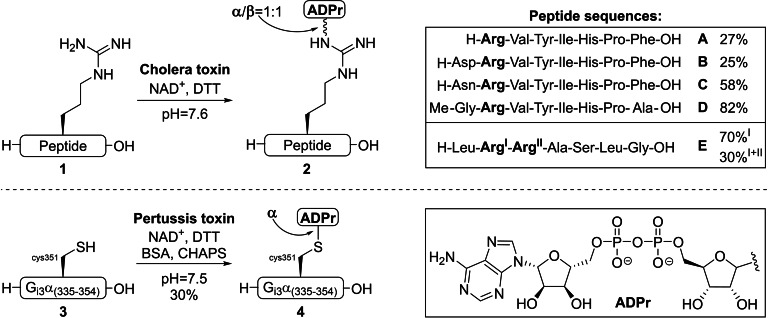
Toxin mediated ADP‐ribosylation of synthetic peptides. Modification of arginine in synthetically derived angiotensine peptides (**1A**–**D**) and kemptide (**1E**) by Kharadia & Graves (Top). ADP‐ribosylation of Cys‐351 in G_i3_α(335–354): H‐VFDRVTDVIIKNNLKE**‐C‐**GLY‐OH (**3**) by Scheuring & Schramm (bottom). BSA=bovine serum albumin, DTT=dithiothreitol, CHAPS=3‐[(3‐cholamidopropyl)dimethylammonio]‐1‐propanesulfonate and NAD^+^=nicotinamide adenosine dinucleotide.

Different laboratories have successfully exploited the (ADP‐ribosyl)transferase activity of bacterial exotoxins, and also porcine brain NADase, for the modification of free amino acids and their derivatives,[^30^, ^118^, ^119^] but the first ADP‐ribosylated oligopeptides have been reported by Kharadia and Graves (Figure 3, top).^[87]^ The CT‐mediated modification of arginine residues in synthetically derived hormones angiotensin II and III (**1B** and **1A** respectively) and analogues thereof (**1C**–**D**), suggested that enzymatic efficiency benefits from hydrophobic residues next to the arginine acceptor site. Nevertheless, the heptapeptide carrying two arginine residues called kemptide (**1E**) proved to be the best substrate and full conversion to give two different products was observed. Tryptic digestion of the isolated products and subsequent mass‐spectrometry analysis of the obtained fragments indicated that the main product was exclusively MARylated on the first arginine reside (Arg^I^, 70 % yield), while the minor product was modified on both arginine side chains (Arg^I+II^, 30 % yield). It is noteworthy that ADP‐ribosylated kemptide **2E** was, in contrast to its unmodified precursor **1E**, a poor substrate for cAMP‐dependent protein kinase. This is in line with previous reports that observed an interplay between protein phosphorylation and ADP‐ribosylation, suggesting a potential regulatory mechanism.[^113^, ^120^] An analogous strategy for the production of mono‐ADPr peptides was developed by Scheuring and Schramm^[114]^ (Figure 3, bottom), after PT was found to modify the cysteine residues of oligopeptides originating from guanine nucleotide‐binding proteins (G‐proteins).[^121^, ^122^] The 20 amino acid carboxyl‐terminus (*C*‐terminus) of the G_i3_α‐subunit **3** was obtained via solid‐phase peptide synthesis (SPPS) and could be converted into ADPr peptide **4**. However, under optimized conditions at 0 °C only a maximum ADP‐ribosylation level of approximately 30 % could be achieved. Extended reaction times and higher concentrations of NAD^+^ did not increase the yield, while incubation at room temperature or 37 °C resulted in NADase activity or inactivation of the toxin, respectively. Finally, extensive proton NMR analysis of the newly derived peptide **4** was applied to confirm the α‐selective introduction of the ADPr modification on cysteine residues by PT.

#### 3.1.2 Serine and Tyrosine

More than two decades after the first enzymatic synthesis of MARylated peptides, Bonfiglio *et al*. developed a chemoenzymatic approach towards peptides ADP‐ribosylated on tyrosine or serine side chains (Table 1).^[113]^ Incubation of the target peptides **5**, containing either a serine or tyrosine modification site, under systematically optimized reaction conditions with the PARP1/HPF1‐protein complex provided PARylated intermediates **6** that were subsequently truncated to the desired MARylated peptides by PARG and isolated in high purity via boronate‐affinity chromatography (Method I). This two‐step procedure allows for the installation of a single ADPr moiety on a wide variety of substrates as was demonstrated with the efficient modification of peptides **5A**–**L** in a scalable manner. However, occasionally sequences were encountered that yielded a complex mixture of peptides, ADP‐ribosylated at different sites, indicating the need for a more elaborate strategy. In this second approach, the off‐target serine residues were orthogonally protected with a phosphate group (**8A**–**B**) to prevent any non‐selective reactions during the two‐step MARylation approach described above. Afterwards, all phosphoserines were completely dephosphorylated by Lambda phosphatase while conserving the previously installed ADPr moieties to provide target structures **10**. With the addition of the phosphate protecting group strategy, an even broader pallet of oligopeptides with ADPr on selected serine or tyrosine residues is now within reach. The resulting array of ADPr peptides were then used as template in the generation of site‐specific as well as pan‐ADPr antibodies that can be used for the visualization of physiological ADPr processes.

**Table 1:** A chemoenzymatic approach towards peptides ADP‐ribosylated on serine (Ser or S) or tyrosine (Tyr or Y) using the PARP1/HPF1 protein complex by Bonfiglio et al. A two‐step procedure (method I) is compatible with a wide variety of sequences. Substrates with off‐target serine residues can be effectively MARylated by implementing phosphate as protecting group (method II). The ADPr acceptor site in each sequence is depicted bold. NAD+=nicotinamide adenine dinucleotide.

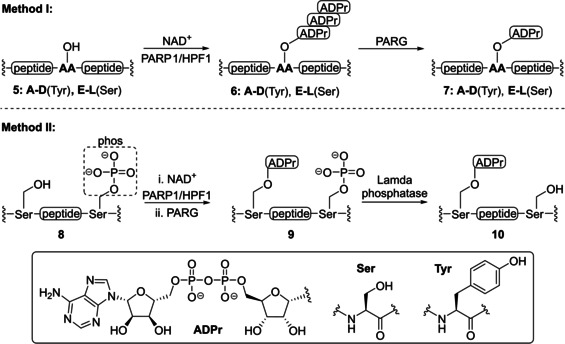			
#	Peptide sequence	Method	Residue
**5A**	Ac‐LRKFYKGKK‐**Y**‐KPLDLRPKKTRAGGK(Biotin)‐NH_2_	I	Tyr
**5B**	Ac‐KNFTK‐**Y**‐PKKFYPLGGK(Biotin)‐NH_2_	I	Tyr
**5C**	Ac‐TGGVKKPHR‐**Y**‐RPGTVALRGGK(Biotin)‐NH_2_	I	Tyr
**5D**	Ac‐LVRHRR‐**Y**‐KHTHGGK(Biotin)‐NH_2_	I	Tyr
**5E**	Ac‐ARTKQTARK‐**S**‐TGGKAPRKQLAGGK(Biotin)‐NH_2_	I	Ser
**5F**	Ac‐ATKAARK‐S^ADPr^‐APATGGVKKPHRYRPGGGK(Biotin)‐NH_2_	I	Ser
**5G**	Ac‐**S**‐GRGKGGKGLGKGGAKRHRGGK(Biotin)‐NH_2_	I	Ser
**5H**	Ac‐KVAKPKKAAK‐**S**‐AAKAVKPGGK(Biotin)‐NH_2_	I	Ser
**5I**	Ac‐KATGAATPKK‐**S**‐AKKTPKKGGK(Biotin)‐NH_2_	I	Ser
**5J**	Ac‐**S**‐GRGKQGGKARAKAKTRSSGGK(Biotin)‐NH_2_	I	Ser
**5K**	Ac‐RLAKSDEPKK‐**S**‐VAFKKTKGGK(Biotin)‐NH_2_	I	Ser
**5L**	Ac‐PKAPKGK‐**S**‐AGREKKVIHPYSRAGGA‐NH_2_	I	Ser
**8A**	Ac‐APRGKS(phos)GAALSKK‐**S^ADPr^**‐KGQVGGK(Biotin)‐NH_2_	II	Ser
**8B**	Ac‐PEPAK‐**S**‐APAPKKGS(phos)KK AVTKAQKKDGKKRKRGGK(Biotin)‐NH_2_	II	Ser

**Scheme 1:**
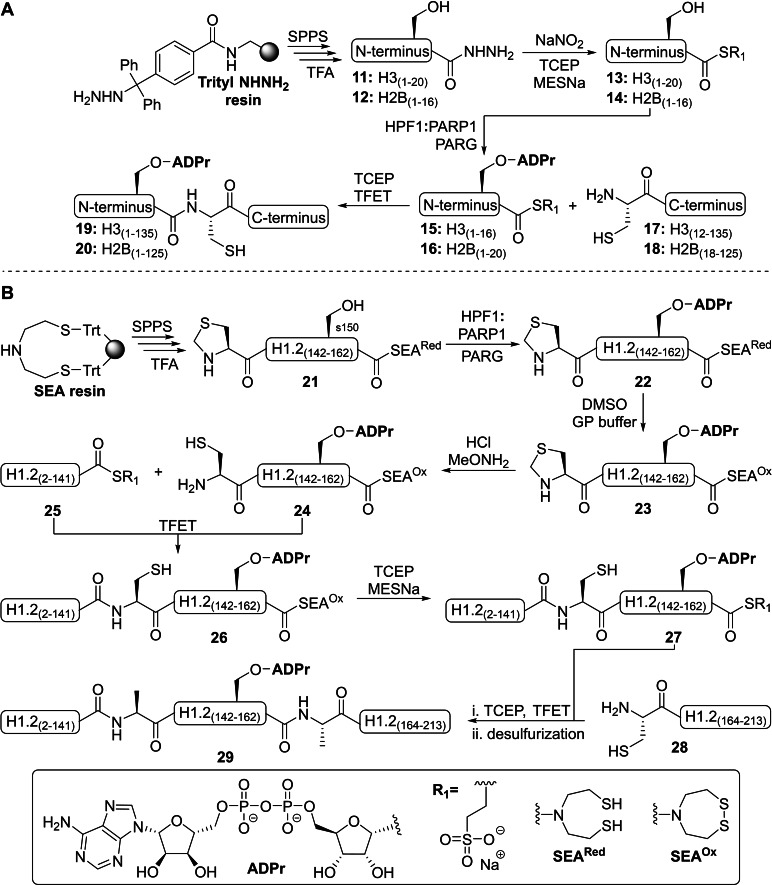
Semisynthesis‐based strategies for the preparation of ADP‐ribosylated serine containing peptides that are compatible with protein ligation reactions by Mohapatra et al. A. Preparation of histones H2B and H3 with an ADP‐modification on the N‐terminal tail (serine 6 and 10 respectively). B. Preparation of histone 1.2 ADP‐ribosylated on Ser‐150, where the synthetic peptide sequences are H3(1–20): H2N‐ARTKQTARKSTGGKAPRKQL‐NHNH2, H2B(1–16): H2N‐PEPKSAPAPKKGSKK‐NHNH2 and H1.2(143–162): Thz‐GGATPKKSAKKTPKKAKKKPA‐SEA. GP buffer=guanidine HCl (6 M)+sodium phosphate (0.1 M). MESNa=sodium 2‐mercaptoethanesulfonate, Thz=thiazolidine, SEA=bis(2‐sulfanylethyl)amide, SPPS=solid‐phase peptide synthesis, TCEP=tris(2‐carboxyethyl)phosphine and TFET=trifluoroethanethiol.

While the previously described procedures are limited to the production of short peptide constructs, Mohapatra *et al*. focused on the development of methodologies that allows for the assembly of full‐length proteins with site‐specific ADPr modifications. To this end, hydrazine functionalized *N*‐terminal tails of histones H3 **11** and H2B **12** were prepared via Fmoc‐based SPPS with *N,N′*‐diisopropylcarbodiimide (DIC)/oxyma‐mediated peptide couplings on a PEG‐resin equipped with an *N*‐tritylhydrazine linker (Scheme 1A).^[123]^ Subsequent conversion of the peptides into thioesters **13** and **14** was effected by sodium nitrite followed by the addition of sodium 2‐mercaptoethanesulfonate (MESNa) and tris(2‐carboxyethyl)phosphine (TCEP). In contrast to the procedure described above, the MARylation of peptides **13** and **14**, on Ser‐10 and Ser‐6 respectively, were realized in a single step by incubation with a cocktail of HPF1, PARP1 and PARG. It is noteworthy that high concentrations of co‐factor HPF1 seem to limit the role of PARP1 as ADPr chain elongator and direct its focus to mono‐ADP‐ribosylation, which is in line with the observation that HPF1 binding causes steric blockage of the elongation/acceptor site of PARP1.^[124]^ A native chemical ligation (NCL) reaction with the corresponding recombinant *C*‐terminal histone fragments **17** or **18** provided the full‐length histones **19** and **20** after purification with semi‐preparative reversed‐phase HPLC. In a nucleosome remodeling assay it was demonstrated that the oligo‐ADP‐ribosylated histones were a much preferred substrated for chromatin remodeler ALC1 in comparison to their respective mono‐ADP‐ribosylated counterparts **19** and **20**. Hereby substantiating the relevance of serine poly‐ADP‐ribosylation in the regulation of the chromatin architecture.

In order to gain access to modification sites throughout the entire protein, Tashiro *et al*. pursued a design that supports modular functionalization at both the *N*‐ and *C*‐terminus of serine ADPr peptides. After serine 150 of linker histone 1.2 was validated as target of HPF1:PARP1 mediated ADP‐ribosylation, synthetic peptide **21** encompassing this modification site was generated on a polystyrene resin, loaded with a bis(2‐sulfanylethyl)amido (SEA)‐linker (Scheme 1B).^[125]^ The *N*‐terminal cysteine residue was protected with a thiazolidine (Thz) in order to prevent enzymatic ADP‐ribosylation as well as nonenzymatic conjugation to free ADP‐ribose. Initially, the use of a *C*‐terminal acyl hydrazide was explored, but conditions required for the thioesterfication of this functionality were incompatible with the Thz protecting group.^[126]^ Enzymatic ADP‐ribosylation of fragment **21** with near quantitative conversion was followed by the oxidation of SEA to give ADPr peptide **23**. The oxidized SEA group is, in contrast to its reduced form, inert to NCL reactions. The *N*‐terminal cysteine was liberated under acidic conditions and conjugated to recombinant H1.2 (2–141) with trifluoroethanethiol (TFET) as thiol catalyst to yield **26**. The reducing agent TCEP was deliberately omitted to preserve the oxidized SEA‐ring and prevent any cross‐linking. Incubation of H1.2 (2–162) **26** with TCEP and MESNa facilitated conversion of the SEA‐moiety into MES‐thioester **27**, which could be coupled to the remaining H1.2 *C*‐terminal segment **28** using similar conditions as before. Finally, H1.2‐S105‐ADPr **29** was obtained through a radical‐based desulfurization to restore ligation junction cysteines into native alanine residues and the whole APPr protein could be efficiently purified by HPLC. Using ADPr‐protein **29**, it was demonstrated that a single serine ADPr modification was sufficient to modulate chromatin condensation dependent on the linker‐histone.

#### 3.1.3 Aspartic and Glutamic Acid

Aimed at finding enzymes that ADP‐ribosylate other residues than serine, Tashiro *et al*. prepared model peptides **30 A**, containing a diverse set of amino acids, to screen a panel of known ADP‐ribosyl transferases (Table 2).^[10]^ The amidated peptides were assembled on a Rink amide resin using DIC/Oxyma‐mediated peptide couplings and purified by reversed‐phase HPLC after cleavage from the resin under acidic conditions. Incubation of peptide **30 A** with NAD^+^ (10 mM) and the catalytic domain of PARP14 at 25 °C effectively led to MARylation of the glutamic acid (**31 A**), as was confirmed by mutational analysis (E9 A) and hydroxylamine treatment.^[66]^ The peptide was still a suitable substrate for PARP14^cat^ after mutation of the glutamate residue to aspartic acid (**30G** to **31G**) and after optimization of the reaction conditions, >50 % conversion of the starting material could be reached within 30 minutes. The observed chemospecificity of PARP14 towards acidic residues is in line with previous findings.^[127]^ A series of peptides (Glu: **30B**–**F** and Asp: **30H**–**J**) were successfully ADP‐ribosylated in milligram quantities under the optimized conditions, which demonstrates that conversion efficiency is independent of the peptide sequence. During LC–MS analysis of the reaction mixture of PARP1 fragment **31F**, different peaks with the desired product mass were observed. This suggests the formation of regioisomers, through acyl migration^[128]^ from the anomeric 1’’‐OH to the 2’’‐ and 3’’‐OH of the distal ribose (Table 2, bottom). The NMR spectra of peptides **31F** and **31J** confirmed that Glu‐ and Asp‐ADPr exist as a mixture of three regioisomers at equilibrium, where 1’’‐*O‐*ADPr is the minor species, even under physiological conditions (PBS buffer, pH=7.4 and 37 °C). Interestingly, in a time‐course assay, ADP‐ribosylated glutamate residues were found to initiate ALC1‐mediated chromatin remodelling with similar efficiency as Ser‐ADPr.

**Table 2:** Chemoselective mono‐ADP‐ribosylation of model peptides on glutamic/aspartic acid, serine or arginine residues by PARP14, HPF1/PARP1 or ARTC2.2, respectively. The modification on Glu and Asp was shown to rapidly migrate to the 2’’‐ and 3’’‐OH positions under physiological conditions. SPPS=solid‐phase peptide synthesis and TFA=trifluoroacetic acid.

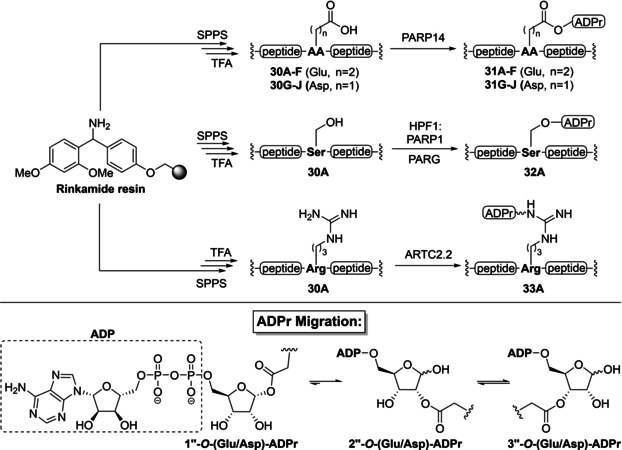			
#	Peptide sequence	Transferase	Residue
**30A**	H‐GWTARKSA**E**VGAGK‐NH_2_	PARP14	Glu
**30B**	H‐GWTARKAA**E**VGAGK‐NH_2_	PARP14	Glu
**30C**	H‐CGWTARKSA**E**HCVGAGK‐NH_2_	PARP14	Glu
**30D**	H‐P**E**PAKSAPAPKKGSKK‐NH_2_	PARP14	Glu
**30E**	H‐S**E**TAPAAPA‐NH_2_	PARP14	Glu
**30F**	H‐AAPV**E**VVAPR‐NH_2_	PARP14	Glu
**30G**	H‐GWTARKSA**D**VGAGK‐NH_2_	PARP14	Asp
**30H**	H‐CGWTARKSA**D**HCVGAGK‐NH_2_	PARP14	Asp
**30I**	H‐GWTAAKSA**D**HCVGAGK‐NH_2_	PARP14	Asp
**30J**	H‐AAPV**D**VVAPR‐NH_2_	PARP14	Asp
**30A**	H‐GWTARK**S**AEVGAGK‐NH_2_	PARP1	Ser
**30A**	H‐GWTA**R**KSAEVGAGK‐NH_2_	ARTC2.2	Arg

Model peptide ADP‐ribosylated on serine **32A** was also prepared as a control compound with the PARP1/HPF1 protein complex in the presence of PARG to suppress unwanted polymerisation (Table 1 and Scheme 1). Interestingly, the screening of enzyme activity showed that the mouse ADPr‐transferase ARTC2.2^[129]^ was capable of selectively modifying the arginine side chain of the model substrate. This resulted in the production of **33A**, and represents an improvement over the arginine ADP‐ribosylation mediated by PT as described by Scheuring and Schramm^[114]^ (see Figure 3). Further analysis revealed that a commercially available pan‐ADP‐ribose detection reagent macrodomain Af1521,^[130]^ has a strong preference for Arg‐linked ADPr peptides over the Asp‐, Glu‐ and Ser‐linked counterparts.^[10]^

### 3.2 Chemical Synthesis of Native ADP‐Ribose Linkages

#### 3.2.1 Asparagine and Glutamine

**Scheme 2:**
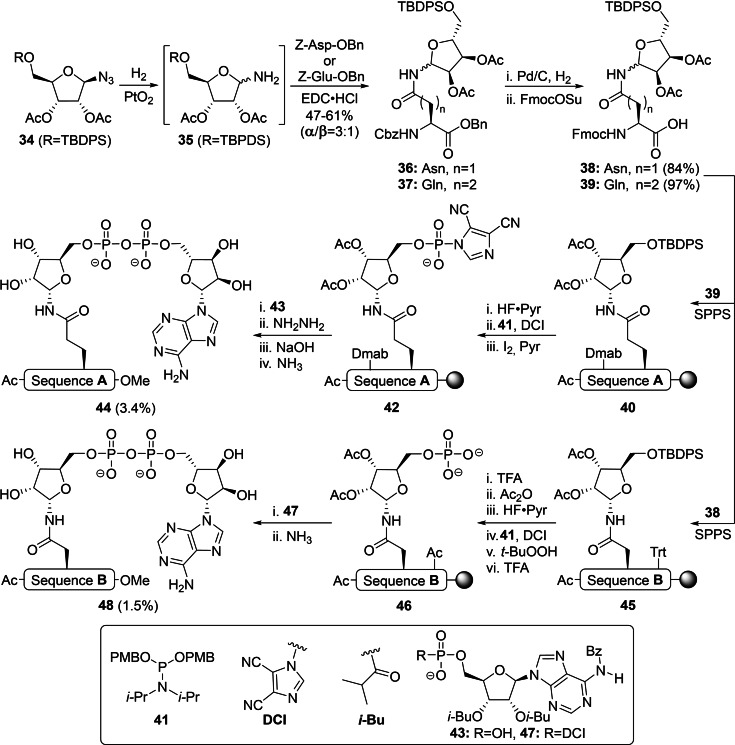
An SPPS‐based approach towards ADP‐ribosylated peptides on asparagine and glutamine by Van der Heden van Noort et al. Sequence A=Ac‐VANIEV‐OMe and sequence B=Ac‐PQPSKSA‐OMe, where the modification site is highlighted (bold). Ac=acetyl, Bz=benzoyl, Bn=benzyl, Dmab=4‐(N‐[1‐(4,4‐dimethyl‐2,6‐dioxocyclohexylidene)‐3‐methylbutyl]amino)benzyl, DCI=dicyanoimidazole, EDC=1‐Ethyl‐3‐(3‐dimethyl‐aminopropyl)carbodiimide, Fmoc=fluorenylmethoxycarbonyl, i‐Bu=iso‐butyryl, Osu=O‐succinimide, Pyr=pyridine, SPPS=solid‐phase peptide synthesis, TBDPS=tert‐butylchlorodiphenylsilane, TEA=triethylamine, TFA=trifluoroacetic acid, Trt=trityl and Z=benzyloxycarbonyl.

The earliest report of fully synthetic approaches towards ADP‐ribosylated peptides was by Van der Heden van Noort *et al*., who prepared ribosylated asparagine **38** and glutamine **39** building blocks via an EDC‐mediated peptide coupling between hemiaminal intermediate **35** and carboxybenzyl (Cbz)‐protected aspartic or glutamic acid respectively (Scheme 2).^[131]^ Successful separation of the individual anomers by silica gel column chromatography was followed by protective group manipulations to provide α‐configured asparagine **38** and glutamine **39** that are compatible with Fmoc‐based SPPS conditions. The latter building block was incorporated in a model hexapeptide (sequence A) using a BOP/HOBt‐coupling procedure on a Tentagel resin equipped with the base‐labile 4‐hydroxymethylbenzoic acid (HMBA) linker. Selective deprotection of the 5’‐OH of immobilized peptide **40** with HF⋅TEA allowed for the on‐resin introduction of the ADPr moiety, which commenced with the 4,5‐dicyanoimidazole (DCI)‐assisted phosphitylation with amidite **41**. The resulting phosphite triester intermediate was oxidized and liberated from *para*‐methoxybenzyl (PMB)‐groups using iodine in pyridine.^[132]^ Addition of suitably protected adenosine monophosphate **43** to phosphorimidazolidate **42** led to construction of the pyrophosphate linkage and the desired glutamine ADPr peptide **44** was generated via a sequence of deprotection steps. First, the Dmab‐moiety on the glutamic acid residue was cleaved via a two‐step procedure comprising hydrazine and NaOH,^[133]^ after which the remaining protecting groups were removed while concurrently liberating the peptide from the resin using methanolic ammonia. Liquid chromatography‐mass spectrometry (LC–MS) analysis of the crude mixture indicated that formation of ADPr peptide **44** was accompanied by the generation of the *C*‐terminal carboxamide, terminal monophosphate and the corresponding H‐phosphonate as side products. Although the desired product could be isolated by extensive HPLC purification, it was hypothesized that formation of the monophosphate and H‐phosphonate contaminations could be reduced by switching the two pyrophosphate precursors. To this extent, asparagine derivative **38** was integrated in a heptapeptide (sequence B) via the same procedure described above to yield peptide **45**. Acid‐labile trityl (Trt)‐groups on the peptide backbone were exchanged for acetyl moieties and the ribofuranoside was desilylated prior to phosphorylation of the 5‐OH with phosphoramidite **41** in the presence of DCI. Oxidation of the phosphite triester intermediate with *t‐*BuOOH and PMB removal under acidic conditions now provided phosphoribosylated peptide **46**. The addition of an excess of adenosine phosphorimidazolidate **47** was expected to counterbalance the partial hydrolysis of the reagent and thus minimize residual phosphate **46**. Unfortunately, after final deprotection and cleavage, similar by‐products were obtained as in the first procedure, which once more emphasized the complex nature of phosphorus chemistry. Regardless of the remaining challenges in the on‐resin construction of ADPr, this approach has enabled the production of well‐defined peptides ADP‐ribosylated on glutamine (**44**) and asparagine (**48**) in acceptable yields and these syntheses provided a benchmark for all ensuing synthetic methodologies.

**Scheme 3:**
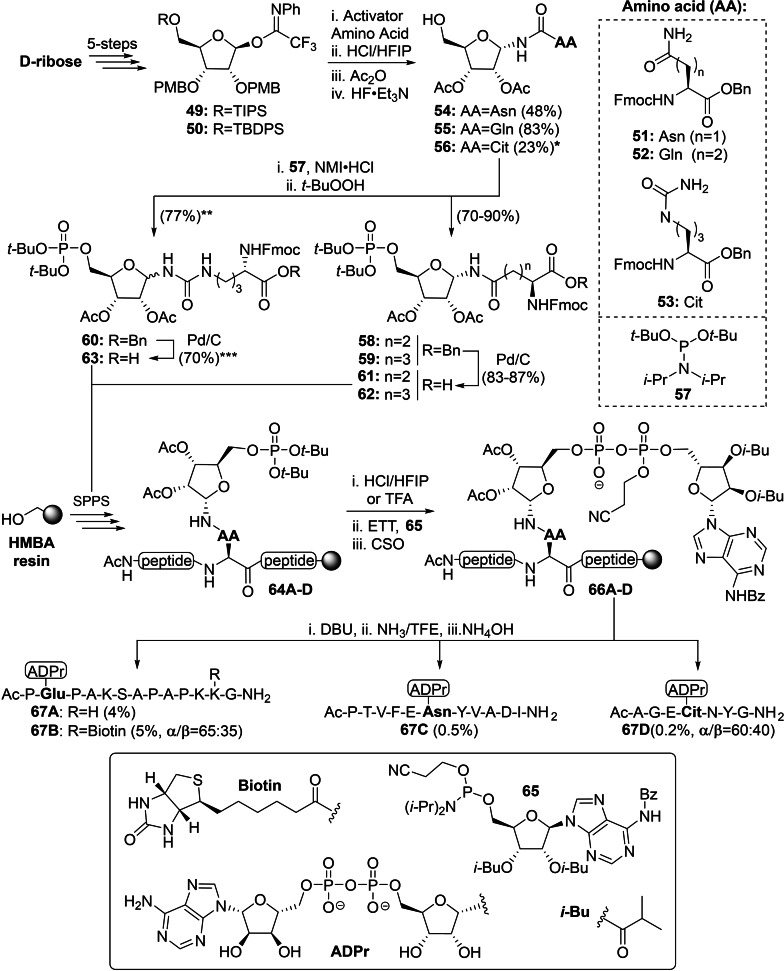
ADP‐ribosylation of asparagine, glutamine and citrulline via an updated methodology by Kistemaker et al. Activator is tert‐butyldimethylsilyl trifluoromethanesulfonate (TBSOTf) for asparagine and citrulline, and HClO4‐SiO2 for glutamine. Citrulline products were obtained as anomeric mixture: *(α/β=34 : 66), **(α/β=60 : 40) and ***(α/β=62 : 38). Ac=acetyl, Asn=asparagine, Bz=benzoyl, Bn=benzyl, Cit=citrulline, CSO=(1S)‐(+)‐(10‐camphorsulfonyl)‐oxaziridine, DBU=1,8‐diazabicyclo‐[5.4.0]undec‐7‐ene, ETT=5‐ethylthiotetrazole, Fmoc=fluorenylmethoxycarbonyl, Gln=glutamine, HFIP=hexafluoro‐iso‐propanol, i‐Bu=iso‐butyryl, NMI=1‐methylimdazole, Ph=phenyl, PMB=para‐methoxybenzyl, SPPS=solid‐phase peptide synthesis, TBDPS=tert‐butylchlorodiphenylsilane, t‐Bu=tert‐butyl,TFE=trifluoroethanol and TIPS=triisopropylsilane.

The efforts of Kistemaker *et al*. have led to the discovery of a stereoselective glycosylation reaction that enabled the construction of α‐ribofuranosyl linkages on various amino acid side chains (including glutamate, aspartate and serine)^[134]^ and was first applied in the synthesis of peptides MARylated on asparagine, glutamine and citrulline (Scheme 3).^[135]^ The required trifluoroacetimidate donors **49** and **50** were derived from D‐ribose in 5 steps and carried non‐participating PMB moieties on the 2‐ and 3‐OH along with an bulky α‐directing triisopropylsilyl (TIPS) or *tert*‐butyldiphenylsilyl (TBDPS) ether on the primary 5‐OH.^[134]^ Ribosylation of asparagine **51** and glutamine **52** ^[134]^ proceeded in an α‐selective manner upon activation of donor **50** with *tert*‐butyldimethylsilyl trifluoromethanesulfonate (TBSOTf) or donor **49** with HClO_4_‐SiO_2_, respectively. Removal of the PMB ethers with catalytic amounts of HCl in hexafluoro‐2‐propanol (HFIP)^[136]^ and subsequent acetylation of the freed 2‐ and 3‐OH positions was followed by desilylation with HF⋅triethylamine (TEA) as fluorine source to provide ribosylated amino acids **54** and **55**. The reaction between donor **50** and the less reactive citrulline **53** mediated by TBSOTf was less selective and provided an anomeric mixture (α/β=78 : 22). While the α‐configured product was isolated and used in the subsequent steps, further isomerization occurred during acidolysis and as a result, furnished functionalized citrulline **56** after acetylation and TBDPS removal as a mixture of anomers (α/β=34 : 66). All three ribosylated amino acids **54**–**56** were phosphorylated prior to SPPS in order to circumvent the troublesome on‐resin formation of phosphomonoesters as described by van der Heden van Noort *et al*.^[131]^ Activation of *t*‐Bu protected phosphoramidite **57** with 1‐methylimdazole hydrochloride and subsequent oxidation of the phosphotriester intermediate with *t*‐BuOOH^[137]^ yielded asparagine **58** and glutamine **59** without affecting the anomeric configuration. Ensuing removal of the benzyl esters by hydrogenolysis afforded the first phosphorylated building blocks **61** and **62** that are compatible with Fmoc‐based SPPS conditions. Since citrulline **56** was configurationally unstable during the two‐step phosphorylation procedure as well as under the applied hydrogenation conditions, building block **63** was used as obtained (α/β=62 : 38) in the following solid‐phase synthesis. The peptide sequences of interest were assembled with phosphoribosylated intermediates **61**–**63** on a Tentagel resin loaded with an HMBA linker using HCTU/DIPEA. A series of protecting group manipulations, analogous to the earlier published procedures (Scheme 2),^[131]^ were required to replace any acid‐labile functionalities on the peptide backbone with acetyl moieties. Although deprotection of the phosphate was preferably executed with 1 equivalent of HCl in HFIP to minimize anomerization, trifluoroacetic acid (TFA) was required for efficient removal of the *t*‐Bu moieties from biotinylated peptide **64B**.

Kistemaker *et al*. then explored an alternative strategy for pyrophosphate synthesis, as initially reported by Gold *et al*., which uses phosphoramidite P(III) chemistry, for the on‐resin construction of the pyrophosphate linkage of ADP‐ribose.^[138]^ Thus, adenosine phosphoramidite **65** was activated with 5‐(ethylthio)‐1H‐tetrazole (ETT) and coupled to the immobilized peptide phosphomonoesters. This was directly followed by oxidation of the P(V)‐P(III) intermediates with CSO to yield protected ADPr peptides **66 A**–**D**. The cyanoethyl (CNE)‐group was cleaved from the pyrophosphate with the non‐nucleophilic base DBU, while the Dmab or *N*‐Allyloxycarbonyl (Alloc) groups, if present, were removed from the peptide side‐chains with hydrazine or Pd(PPh_3_)_4_, respectively. A mixture of ammonia in trifluoroethanol (TFE) was found to be superior to methanolic ammonia for liberation of the peptides from the resin and exclusively provided carboxamide products. The addition of NH_4_OH in the final step was required to ensure the complete removal of the benzoyl group from the exocyclic amine of adenosine. The oligopeptides ADP‐ribosylated on glutamine **67A** (R=H, 4 %) and **67B** (R=biotin, 5 %, α/β=65 : 35) were obtained in high purity through a combination of HPLC and boronate affinity chromatography. The anomeric ratio of the biotinylated analogue **67B** clearly demonstrated the impact of the acidic deprotection conditions (5 % TFA) on the isomerization of the N‐glycosidic linkage. ADPr‐Asn **67C** (obtained in 0.5 %) and ADPr‐Cit **67D** (isolated in 0.2 %, α/β=60 : 40) were purified with anion‐exchange chromatography and the low yield of the latter two peptides was attributed to premature cleavage of the peptide during hydrazine treatment. The binding selectivity of catalytically inactive macrodomains MacroD2^TM^ and TARG1^D125A^ was evaluated with ADPr‐peptides **67A**, **67C** and **67D**. The former did not exhibit a clear substrate specificity and bound all substrates, while the latter showed no affinity towards peptide **67A**.

#### 3.2.2 Serine, Threonine and Cysteine

**Scheme 4:**
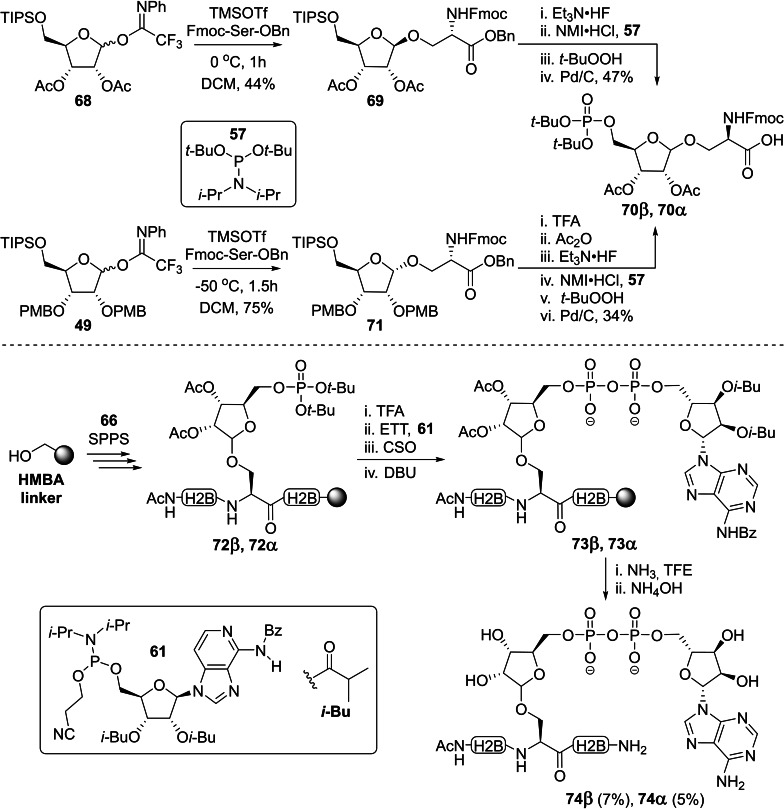
Synthesis of an H2B_4–14_ peptide fragment (full sequence=AcNH‐PAK**S**APAPKKG‐NH_2_) with an α‐ or β‐configured ADP‐ribosylated serine residue by Voorneveld *et al*. Ac=acetyl, Bn=benzyl, Bz=benzoyl, CSO=(1S)‐(+)‐(10‐camphorsulfonyl)‐oxaziridine, DBU=1,8‐diazabicyclo[5.4.0]undec‐7‐ene, ETT=5‐ethylthiotetrazole, Fmoc=fluorenylmethoxycarbonyl, *i‐*Bu=*iso‐*butyryl, PMB=*para*‐methoxybenzyl,NMI=1‐methylimdazole, Ph=phenyl, SPPS=solid‐phase peptide synthesis, *t*‐Bu=*tert*‐butyl, TFA=trifluoroacetic acid, TFE=trifluoroethanol, TIPS=triisopropylsilane and TMSOTf=trimethylsilyl trifluoromethanesulfonate.

Voorneveld *et al*. expanded the exploratory work of Kistemaker *et al*. on the α‐selective trifluoroacetimidate‐based ribosylation of serine by preparing a β‐directing donor (Scheme 4).^[9]^ *N*‐Phenyl trifluoro acetimidate donor **68** could be prepared from D‐ribose in a similar fashion as the known α‐selective donor **49**, but was equipped with acetyl groups instead of non‐participating PMB ethers on the 2‐ and 3‐OH. Upon activation of the donor with trimethylsilyl trifluoromethanesulfonate (TMSOTf), the highly reactive oxocarbenium species that is formed upon activation of the donor with TMSOTf can be stabilized by the formation of a dioxolenium ion using the 2‐OH acetyl moiety in a process referred to as neighboring group participation. As a result, attack by a nucleophile on the β‐face of the ribose ring is highly favored. Acetyl migration to the serine aglycon^[139]^ could be minimized by optimization of the donor/activator ratio and reaction temperature, and β‐ribosylated serine **69** could be obtained in 55 % yield. Deprotection of the TIPS group with HF⋅TEA and the previously reported two‐step phosphorylation procedure^[137]^ using *t*‐Bu protected amidite **57** was followed by hydrogenolysis to provide SPPS‐building block **70β**. The α‐selective introduction of Fmoc‐Ser‐OBn on donor **49** proceeded uneventfully,^[134]^ but initial attempts to remove the PMB‐ethers using varying concentrations of HCl in HFIP resulted in degradation of the *O*‐glycosidic linkage. Fortunately, no side reactions were observed upon exposure of **71** to an excess of TFA and the freed 2‐ and 3‐OH could be acetylated with acetic anhydride in pyridine without intermediate purification. The same sequence of reaction conditions described for the β‐anomer could then be applied to provide its α‐counterpart **70α**. Incorporation of building blocks **70β** and **70α** in hendecapeptide, originating from the *N*‐terminus of histone H2B, and on‐resin construction of the ADPr moiety based on the P(V)‐P(III) coupling were established by adopting the protocol described by Kistemaker *et al*. (Scheme 3).^[135]^ Cleavage from the resin using ammonia in TFE and a final deprotection with NH_4_OH furnished the desired serine ADP‐ribosylated peptides **74β** and **74α** after HPLC purification in 7 % and 5 % yield, respectively. The stability of these two anomers to ARH3 was evaluated in an ADP‐ribosyl hydrolase assay, which showed that only the α‐anomer was sensitive to the enzyme‐catalyzed hydrolysis. Thus, it has been unmistakably confirmed that the naturally occurring glycosidic Ser‐ADPr linkage is α‐configured.

**Scheme 5:**
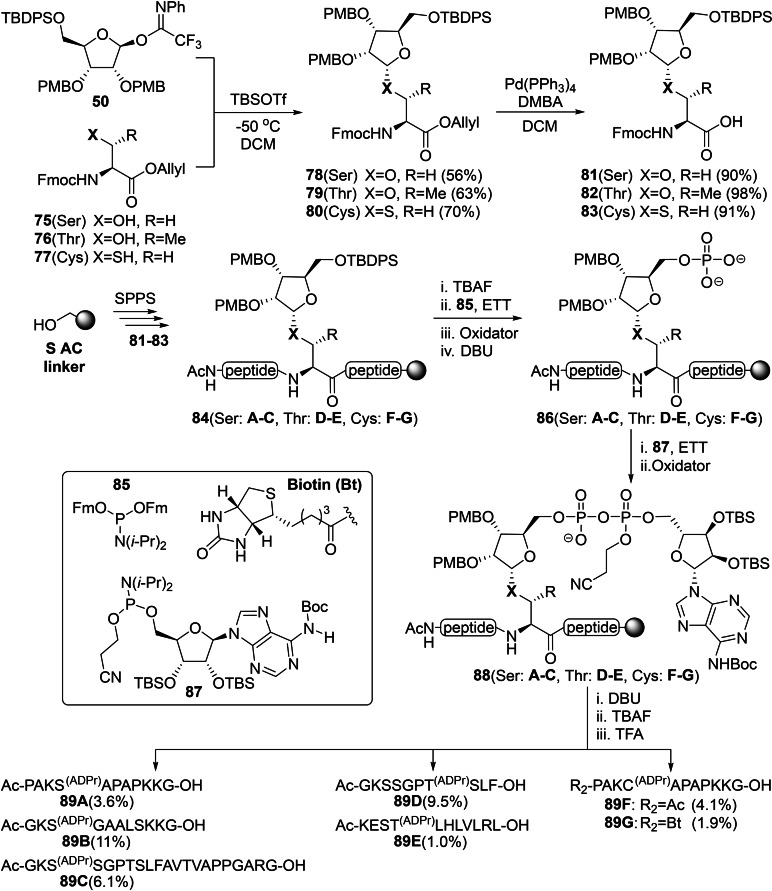
An improved and versatile methodology for synthetic oligopeptides ADP‐ribosylated on serine, threonine or cysteine side chains by Voorneveld *et al*. CSO was a suitable oxidation agent for peptides **A**–**F**, while the milder *t*‐BuOOH was used for biotinylated peptide **G**. Ac=acetyl, Boc=*tert*‐butyloxycarbonyl, CSO=(1S)‐(+)‐(10‐camphorsulfonyl)‐oxaziridine, DBU=1,8‐diazabicyclo[5.4.0]undec‐7‐ene, DMBA=1,3‐dimethylbarbituric acid, ETT=5‐ethylthiotetrazole, Fm=9‐fluorenylmethyl, Fmoc=fluorenylmethoxycarbonyl, Ph=phenyl, PMB=*para*‐methoxybenzyl, SPPS=solid‐phase peptide synthesis, *i‐*Pr=*iso‐*propyl, TBAF=tetrabutylammonium fluoride, TBDPS=*tert*‐butylchlorodiphenylsilane, TBS=*tert*‐butylsilyl, and TFA=trifluoroacetic acid.

The SPPS‐based approach using the HMBA‐resin described above has facilitated the production of peptide fragments ADP‐ribosylated on asparagine, glutamine, citrulline (Scheme 3) and serine residues (Scheme 4), but is limited to *C*‐terminal carboxamide products and includes an extensive amount of protecting group shuffling on the peptide side chains in case of residues such as threonine, serine and glutamic acid. These drawbacks have been addressed in the improved methodology developed by Voorneveld *et al*., for which a series of α‐ribosylated amino acids **78**–**80** were constructed through the coupling of known imidate donor **50** with allyl ester derivatives of serine (**75**), threonine (**76**) and cysteine (**77**) (Scheme 5).^[115]^ The convenient palladium(0)‐catalyzed deprotection of the allyl ester in the presence of 1,3‐dimethylbarbituric acid (DMBA) as allyl cation scavenger furnished building blocks **81**–**83** from their respective precursor in high yields. In contrast to the previous procedures, phosphomonoester formation was postponed until after SPPS and a highly acid‐sensitive S AC linker was selected to provide *C*‐terminal carboxylic acids after cleavage, to more closely resemble naturally processed peptides. Building blocks **81**–**83** were incorporated in various peptide sequences that have been identified in proteomics studies[^140^, ^141^] through HCTU/DIPEA‐assisted condensation on Tentagel resin pre‐loaded with glycine via the previously mentioned S AC linker. Tetrabutylammonium fluoride (TBAF) was found to most efficiently facilitate TBDPS removal of ribosylated peptides **84**, with full conversion taking place in less than 30 min, while HF⋅TEA and HF⋅pyridine required overnight shaking. Phosphoramidite **85**, decorated with base‐labile 9‐fluorenylmethyl (Fm) protecting groups, was coupled to the liberated primary 5‐OH, by activation with ETT, and this was followed by CSO‐mediated oxidation of the phosphite triester intermediate. The use of the methylsulfonylethyl (Mse) protecting group was investigated as an alternative to the Fm‐functionality, but the latter was preferred due to its more efficient deprotection by DBU. Construction of the pyrophosphate linkage was executed according to a two‐step procedure analogous to the 5‐OH phosphorylation to yield fully protected ADPr peptides **88**. Removal of the CNE moiety from the pyrophosphate and ensuing desilylation of the proximal ribose was followed by the simultaneous deprotection and cleavage from the resin under acidic conditions (10 % TFA). The thiol‐based scavenger ethane dithiol (EDT) was added to the cleavage cocktail for ADP‐ribosylated cysteine peptides to suppress migration of the PMB‐cation to the cysteine aglycon. Finally, preparative HPLC purification provided three distinct ADP‐ribosylated serine peptides (**89A**–**C**), along with two fragments modified on threonine (**89D**–**E**) as well as ADPr‐Cys (**89F**). The *N*‐terminal biotin functionality, to facilitate streptavidin enrichment experiments, of ADPr‐Cys **89G** was found to be oxidized during CSO treatment. Fortunately, the chemoselective oxidation of the P^III^‐intermediates could be achieved with the milder oxidizing agent *t*‐BuOOH which enabled isolation of the final peptide. The synthetic mono‐ADP‐ribosylated peptides were subjected to a collection of ADP‐ribosyl hydrolases.^[115]^ The results showed that the reversal of Cys‐ADPr is not achieved by ARH3. Further screening revealed the *Streptococcus pyogenes* encoded protein SpyMacroD as the first (ADP‐ribosyl)‐hydrolase for cysteine residues.

**Scheme 6:**
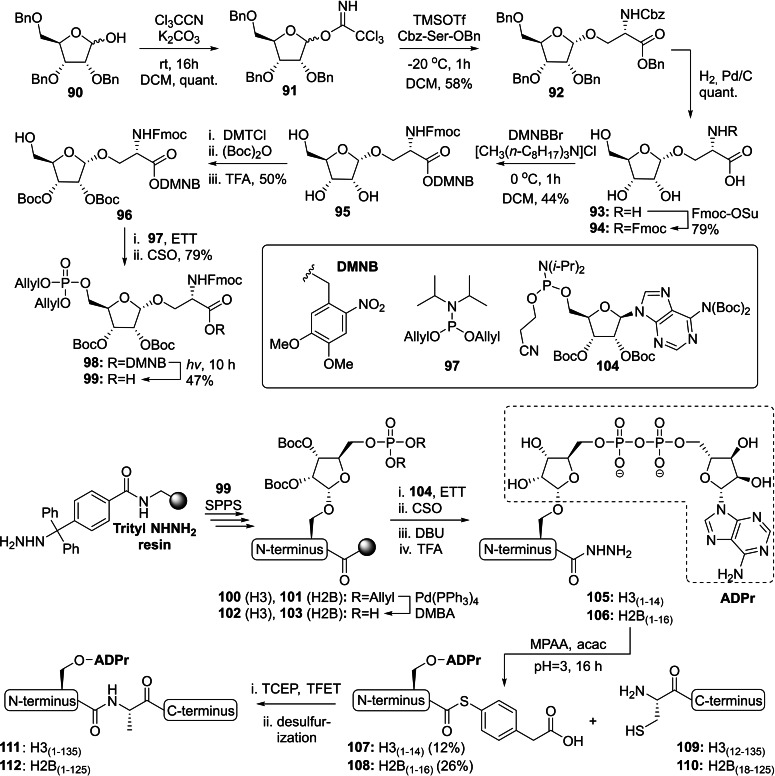
A chemical methodology for the synthesis of full length histones H3 and H2B ADP‐ribosylated on serine 10 and 6, respectively. The N‐tail fragments were obtained via solid‐phase peptide synthesis with key building block **70** and conjugated to the respective truncated recombinant C‐terminus through native chemical ligation. Sequence of synthetic fragments H3(1–14)=ARTKQTARK**S**TGGK and H2B(1–16)=PEPAK**S**APAPKKGSKK. Acac=acetylacetone, Bn=benzyl, Boc=*tert*‐butyloxycarbonyl, Cbz=benzyl chlorocarbamate, CSO=(1S)‐(+)‐(10‐camphorsulfonyl)‐oxaziridine, DBU=1,8‐diazabicyclo[5.4.0]undec‐7‐ene, DMBA=1,3‐dimethylbarbituric acid, DMNB=4,5‐dimethoxy‐2‐nitrobenzyl, DMTCl=4,4‐dimethoxytritylchloride, ETT=5‐(Ethylthio)‐1H‐tetrazole, Fmoc=fluorenylmethoxycarbonyl, *i‐*Pr=*iso‐*propyl, Me=methyl, MPAA=4‐Mercaptophenylacetic acid, Ph=phenyl, SPPS=solid‐phase peptide synthesis, TCEP=tris(2‐carboxyethyl)phosphine, TFET=trifluoroethanethiol and TFA=trifluoroacetic acid.

An alternative strategy to generate ribosylated serine building blocks compatible with Fmoc‐based SPPS was published by Hananya *et al*., who continued to implement these building blocks in the very first synthesis of full‐length ADP‐ribosylated histones via a traceless NCL for the very first time.^[142]^ The synthesis commenced with 2,3,5‐*O*‐benzylated riboside **90** ^[143]^ that was converted into trichloroamidate **91** via treatment with commercially available trichloroacetonitrile under basic conditions (Scheme 6). Activation with a catalytic amount of TMSOTf provided an anomeric mixture (α/β=3 : 1) that could be seperated by silica gel column chromatography to yield the desired α‐anomer **92** in a 58 % yield. A series of protecting group manipulations followed to give **96** that was adequately equipped for the phosphitylation with allyl‐protected phosphoramidite **97** and subsequent oxidation with CSO to yield phosphotriester **98**. Liberation of the carboxylic acid from the photolabile 4,5‐Dimethoxy‐2‐nitrobenzyl (DMNB) group was realized upon UV radiation (370 nm), after which key intermediate **99** could be incorporated into the *N*‐terminal fragment of histone 3 (**100**) and histone 2B (**101**) using standard SPPS conditions. The on‐resin deprotection of the phosphate allyl esters with palladium tetrakis in the presence of DMBA was followed by a sequence of reactions to finalize the ADPr modification. At first, a P(V)‐P(III)^[138]^ coupling between phosphates **101** and **103** with adenosine phosphoramidite **104** was catalyzed by ETT and the resulting intermediate was immediately oxidized with CSO. The cyanoethyl group on the pyrophosphate linkage was removed with DBU and a final treatment with TFA facilitated global deprotection of the acid‐labile Boc‐groups and concomitant cleavage from the trityl resin to yield Ser‐ADPr hydrazine functionalized peptides **105** and **106**. Since the conventional conversion of a hydrazine into a thioester through treatment with nitrous acid^[126]^ is not compatible with the exocyclic amine of adenosine, a milder alternative was explored. The peptide hydrazide was treated with acetylacetone (acac) to form an acyl pyrazole intermediate that further reacts with 4‐mercaptophenyl acetic acid (MPAA) to yield the corresponding thioesters **107** (12 %) and **108** (26 %).^[144]^ The synthetically derived, and MARylated, *N*‐terminal thioesters **107**–**108** were then coupled to their respective recombinantly derived *C‐*terminus **109**–**110** via a NCL in the presence of TCEP and TFET. A final radical‐initiated desulfurization of the ligation site was performed to yield the native full‐length histones H3 (**111**) and H2B (**112**). The use of these newly derived ADP‐ribosylated histones in histone reconstitution assays indicated that ADP‐ribosylation on serine‐6 of H2B (**112**) suppressed chromatin folding and higher‐order reorganization. An effect that was even stronger for ADP‐ribosylated H3 (**111**). Furthermore, treatment of the latter modified histone with a collection of lysine methyltransferases revealed that there is a context dependent interplay between ADP‐ribosylation and methylation of histones.

**Scheme 7:**
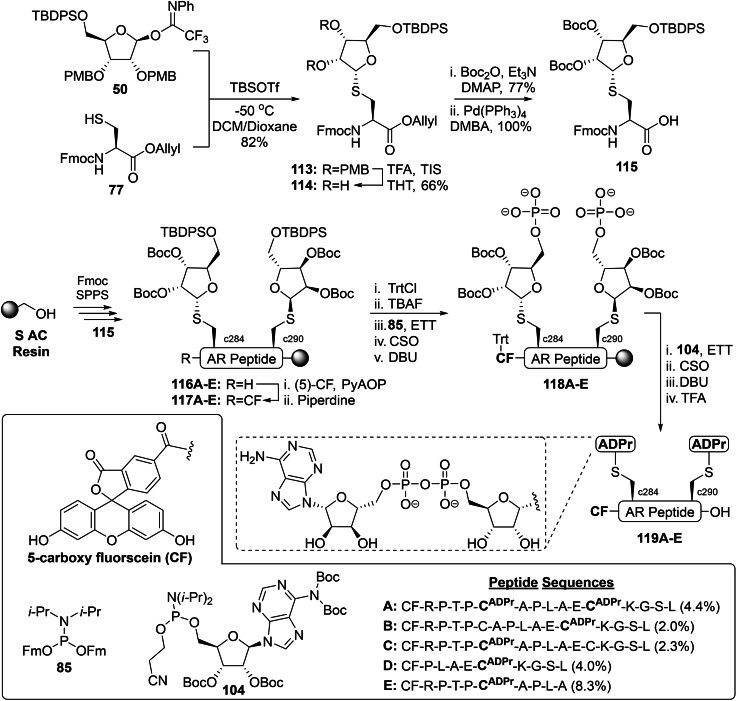
The first approach towards a tandem Cys‐ADP‐ribosylated oligopeptide derived from the androgen receptor (AR) by Wijngaarden *et al*. Boc=*tert*‐butyloxycarbonyl, CSO=(1S)‐(+)‐(10‐camphorsulfonyl)‐oxaziridine, DBU=1,8‐diazabicyclo[5.4.0]undec‐7‐ene, DMAP=4‐Dimethylaminopyridine, ETT=5‐ethylthio‐tetrazole, Fm=9‐fluorenylmethyl, Fmoc=fluorenylmethoxycarbonyl, *i*‐Pr=*iso‐*propyl, Ph=phenyl, PMB=*para*‐methoxybenzyl, PyAOP=(7‐azabenzotriazol‐1‐yloxy)tripyrrolidinophosphonium hexafluorophosphate, TBDPS=*tert*‐butylchlorodiphenylsilane, Tf=trifluoromethanesulfonyl, TFA=trifluoroacetic acid, THT=tetrahydrothiophene, TIS=triisopropylsilane, and Trt=trityl.

The methodology to synthesize peptides ADP‐ribosylated on cysteine of Voorneveld *et al*. was further optimized by Wijngaarden *et al*. to realize the first dual cysteine‐ADP‐ribosylated peptides and assess their relevance in the binding of a PARP9 protein complex (Scheme 7).^[145]^ The α‐selective glycosylation reaction with donor **50** was, in contrast to the original method (Scheme 5), performed with the addition of 10 v/v% dioxane to the DCM mixture to improve the solubility of the cysteine acceptor **77** and increase the reaction rate. Migration of the 2’’‐*O*‐ and 3’’‐*O*‐PMB ethers to the cysteine side chain during the final TFA cleavage was prevented by removing them at the stage of building block **113**. A series of cation scavengers to suppress the PMB migration were investigated. EDT, thioanisole and dimethylsulfide (DMS) gave inconsistent results, but 30 v/v% TFA in tetrahydrothiophene (THT) provided the desired diol **114** in 66 % yield. The synthesis of key intermediate **115** was finalized after a Boc‐protection of the secondary alcohol and deprotection of the allyl ester with tetrakis(triphenylphosphine)palladium in the presence of DMBA.

Conventional Fmoc‐based SPPS chemistry with DIC/Oxyma couplings was applied to assemble a fragment of the *N*‐terminal domain of the androgen receptor (AR) on the acid‐sensitive S AC resin, yielding the (double) ribosylated immobilized peptides **116A**–**E**. The *N*‐terminus was then functionalized with (5)‐carboxy fluorescein (CF) using (7‐azabenzotriazol‐1‐yloxy)‐tripyrrolidinophosphonium hexafluorophosphate (PyAOP)^[146]^ and treated with 20 v/v% piperidine to remove unwanted esters formed on the phenolic alcohols of CF.^[147]^ The freed CF phenols of intermediates **117A**–**E** were then tritylated to avoid unwanted phosphorylation of the CF residue. Desilylation with TBAF primed the construct for the two‐step phosphorylation procedure. Fm‐phosphoramidite was activated by an equimolar amount of ETT and installed on the primary alcohol, resulting in a P(III)‐intermediate that was oxidized to the P(V)‐phosphotriester using CSO. Deprotection of the Fm‐groups by DBU provided the required precursors **118A**–**E** for pyrophosphate construction through a P(V)‐P(III) coupling with Boc‐protected adenosine phosphoramidite **104** under the aegis of ETT.^[138]^ Similarly, the phosphite was oxidized with CSO and the resulting partially protected pyrophosphate was liberated from the 2‐cyanoethyl group using DBU. Since the *S*‐glycosidic linkage is highly acid‐resistant, the original procedure could be further improved by increasing the amount of TFA in the cleavage cocktail from 10 to 50 %. Not only did this result in shorter deprotection times, but it also facilitated cleavage of the relatively acid‐stable 2,2,4,6,7‐pentamethyldihydrobenzofuran‐5‐sulfonyl group (Pbf) from the arginine residues. Reversed‐phase HPLC purification furnished the tandem Cys‐ADP‐ribosylated peptide **119A** in 4.4 % yield along with its MARylated analogues **119B**–**E** in an overall yield varying from 2.0 to 8.3 %. The study further investigated the significance of a tandem Cys‐ADPr modification of AR fragments in the binding with PARP9/DTX3 L protein complex.^[145]^ It turned out that peptide **119A**, which contains the Cys‐ADPr tandem, has a significantly higher affinity than the singly MARylated controls **119B**–**E** and that oligomerization of the PARP9/DTX3 L protein complex was crucial for the interaction.

#### 3.2.3 Aspartic and Glutamic Acid

**Scheme 8:**
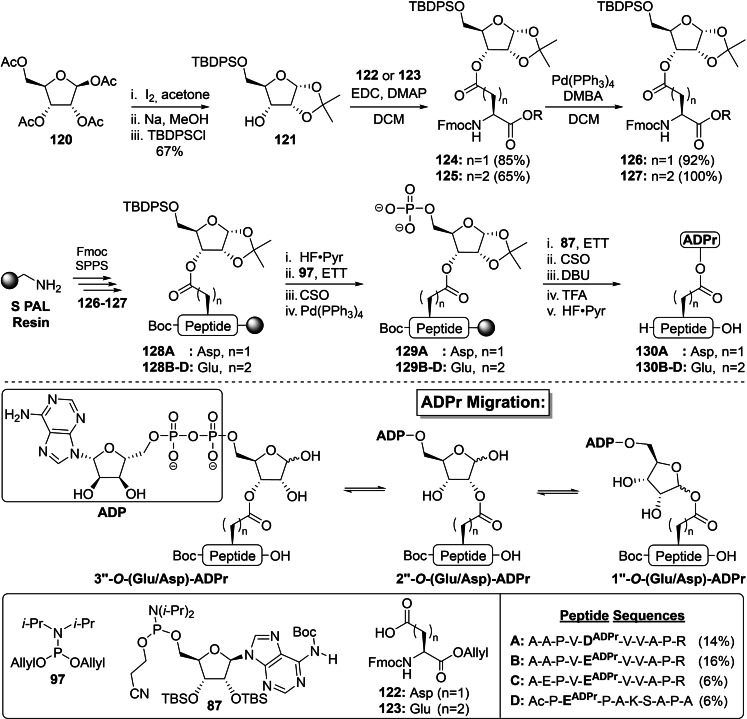
Exploitation of a Steglich esterification in the preparation of oligopeptides ADP‐ribosylated on glutamic or aspartic acid residues. The aspartic and glutamic acid esters migrate between the 1’’‐, 2’’‐ and 3’’‐OH after removal of the isopropylidene functionality. Ac=acetyl, Boc=*tert*‐butyloxycarbonyl, CSO=(1S)‐(+)‐(10‐camphorsulfonyl)‐oxaziridine, DBU=1,8‐diazabicyclo[5.4.0]undec‐7‐ene, DMBA=1,3‐Dimethylbarbituric acid, DMAP=4‐Dimethylaminopyridine, EDC=1‐ethyl‐3‐(3‐dimethylaminopropyl)carbodiimide, ETT=5‐ethylthio‐tetrazole, Fm=9‐fluorenylmethyl, Fmoc=fluorenylmethoxycarbonyl, Me=methyl, *i‐*Pr=*iso‐*propyl, Pyr=pyridine TBDPS=*tert*‐butylchlorodiphenylsilane and TFA=trifluoroacetic acid.

Since the ADPr modification on acidic residues is prone to acyl migration, with the equilibrium leaning towards the acylated 2’’‐ and 3’’‐hydroxyl groups,[^10^, ^128^] Wijngaarden *et al*. devised a fully synthetic route towards peptides ADP‐ribosylated on glutamate and aspartate based on the 3‐*O*‐linked ribosylated building blocks **126** and **127** (Scheme 8).^[10]^ Starting from commercially available tetraacetyl‐*D*‐ribose **120**, an iodine‐promoted acetonide formation^[148]^ and subsequent deacetylation of the remaining 3‐ and 5‐protecting groups was followed by the regioselective introduction of the bulky TBDPS silyl ether to yield precursor **121** in a 67 % overall yield. An EDC‐mediated Steglich esterification with either allyl‐protected aspartic (**122**) or glutamic acid (**123**) derivatives provided intermediates **124** and **125**, respectively, in satisfactory yields of 65–85 %. The synthesis of SPPS‐compatible building blocks **126** and **127** was finalized by the deprotection of the allyl ester functionality with tetrakis(triphenylphosphine)‐palladium in the presence of the scavenger DMBA.

The freshly obtained building blocks **126**–**127** were then incorporated in a set of four peptide fragments, originating from PARP1 and H2B, on Tentagel S PAL resin through DIC/Oxyma‐promoted peptide couplings. The resulting immobilized intermediates **128 A** (Asp) and **128B**–**D** (Glu) were then desilylated using HF‐pyridine followed by a two‐step phosphorylation sequence with allyl‐protected phosphoramidite **107**. First, the free primary alcohols were treated with **97** in the presence of ETT as an activator and the resulting P(III)‐intermediates were immediately oxidized by CSO to the phosphotriesters. Pd(0)‐mediated deprotection of the phosphates furnished intermediates **129A**–**D**, which allowed for the installation of the ADPr moieties through a P(V)‐P(III) pyrophosphate construction.^[138]^ To this end, adenosine phosphoramidite **87** was activated by ETT, coupled to the liberated phosphate and subsequent oxidation yielded the partially protected ADPr constructs. The 2‐cyanoethyl group on the pyrophosphates was eliminated by DBU and a global deprotection step under mild acidic conditions (2.5 % TFA) with concomitant cleavage from the resin resulted in the ADPr peptides carrying TBS functionalities on the 2’‐ and 3’−OH positions. The silylated ADPR‐peptides were isolated using reversed‐phased HPLC and treated with HF‐pyridine. An additional final HPLC purification provided the desired ADPr‐Asp (**130A**) and ADPr‐Glu (**130B**–**D**) peptides in yields up to 16 %. The NMR spectra of the synthetically derived ADPr peptides were in full correspondence to their enzymatically obtained counterparts (Table 2) and showed a mixture of regio‐ and stereoisomers, thus substantiating the inevitability of acyl migration of Glu‐ and Asp‐ADPr.^[10]^

#### 3.2.4 Arginine and Histidine

**Scheme 9:**
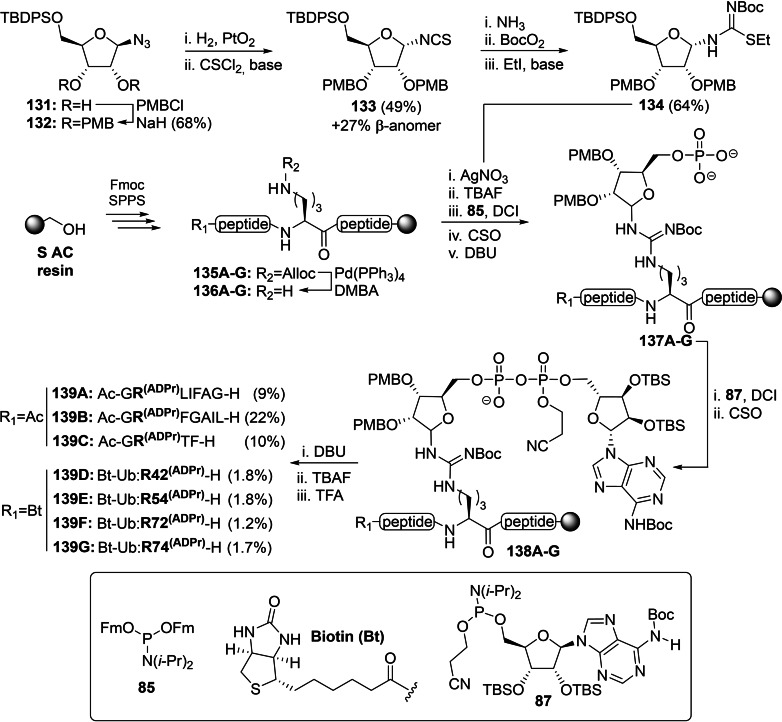
The use of a Lewis acid promoted conjugation between ornithine and isothiourea in the synthesis of arginine‐linked ADPr oligopeptides and full‐length ubiquitin as reported by Voorneveld *et al*. Alloc=*N*‐allyloxycarbonyl, Boc=*tert*‐butyloxycarbonyl, CSO=(1S)‐(+)‐(10‐camphorsulfonyl)‐oxaziridine, DBU=1,8‐diaza bicyclo[5.4.0]undec‐7‐ene, DMBA=1,3‐dimethylbarbituric acid, Et=ethyl, *i‐*Pr=*iso*‐propyl, PMB=*para*‐methoxybenzyl, SPPS=solid‐phase peptide synthesis, TBAF=tetrabutylammonium fluoride, TBDPS=*tert*‐butylchlorodiphenylsilane, TFA=trifluoroacetic acid and Ub=ubiquitin.

The basic nature of the guanidinium moiety of arginine causes, similar to histidine, difficulties in (Lewis) acid‐catalyzed glycosylation reactions, so an alternative route towards Arg‐ADPr was devised by Voorneveld *et al*. who exploited a Lewis acid promoted addition of an ornithine to an isothiourea riboside (Scheme 9).^[116]^ To this extent, full protected β‐azido‐ribofuranoside **132** was derived from commercially available ribofuranose tetraacetate in 4 steps.^[149]^ A Pt(IV)‐catalyzed reduction of the anomeric azide resulted in the highly labile ribosylamine intermediate that was filtered over a pad of celite and immediately converted into an anomeric mixture of isothiocyanate **133** using thiophosgene. The obtained anomers could be conveniently separated by silica gel column chromatography and the α‐anomer was subjected to the following three‐step procedure. First, a thiourea intermediate was produced via ammonolysis, followed by protection of the newly introduced amine with the *tert*‐butyloxycarbonyl (Boc) group and finally formation of the desired isothiourea **134** through alkylation of the sulfur atom with ethyl iodide. In order to test and optimize on‐resin ADP‐ribosylation, various model peptides **135A**–**C** were assembled on Tentagel resin equipped with an acid‐labile S AC linker with an orthogonal alloc‐protected ornithine installed at the modification site. After selective deprotection of the Alloc‐functionality with a palladium(0) catalyst, on‐resin guanidinylation of the immobilized ornithine constructs **136A**–**C** could be efficiently established in the presence of silver nitrate as Lewis acid. Desilylation of the 5‐OH with TBAF proceeded uneventfully, but the subsequent phosphorylation reaction using the phosphoramidite procedure was hampered by phosphitylation of the guanidine group. Optimization of both the activator and amount of Fm‐protected amidite **85**, revealed that DCI was superior over tetrazole and ETT in terms of selectivity and that unwanted guanidine phosphitylation could be further suppressed by reducing the original 5 equivalents to a 2.5‐fold excess of the phosphoramidite. Ensuing deprotection of the Fm‐groups using DBU provided phosphomonoester intermediates **137**, primed for the reaction with adenosine phosphoramidite **87** through the established P(V)‐P(III) coupling. Identical deprotection and cleavage conditions as described above (Scheme 5) were applied to obtain ADPr‐Arg peptides **139A**–**C** after preparative HPLC in yields of 9–22 %. Spectroscopic analysis indicated that the final products were obtained as anomeric mixture (α/β=6 : 4), which is in accordance with observations of spontaneous anomerization under neutral or acidic conditions made by Oppenheimer *et al*.^[30]^

Next, it was demonstrated that the silver‐mediated guanidinylation methodology is not limited to oligopeptides, but is also applicable for the functionalization of whole proteins (Scheme 9). Full‐length ubiquitin analogues **135D**–**G** were synthesized using SPPS with Arg‐42, −54, −72 or −74 replaced with Alloc‐protected ornithine respectively.^[116]^ All four proteins were ADP‐ribosylated without major adjustments. However, a significantly higher concentration of TFA (90 v/v% versus the original 10 v/v% used for peptides **139A**–**C**) was required to ensure complete removal of the Pbf protective groups from the remaining arginine residues. Interestingly, no degradation of the pyrophosphate or the N‐glycosidic bond was observed by LC–MS after prolonged reaction times of up to 1.5 h, suggesting that ubiquitin somehow stabilizes the modification. ADP‐ribosylated ubiquitin proteins **139D**–**G** were successfully isolated by HPLC in a 1.2–1.8 % yield, but were contaminated with varying amounts (14–30 mol %) of their respective phosphoribosylated precursor. The observed stability of ADP‐ribosylated ubiquitin under acidic conditions suggests that the formation of this side product is the result of the incomplete coupling of phosphates **137D**–**G** to adenosine amidite **87** and not due to acid‐promoted degradation of ADPr. In hydrolysis and ligation assays with Legionella enzymes (DupA and SdeA), it was demonstrated that Arg‐ADPr peptides **139A**–**C** as well as MARylated Ubiquitin **139D**–**F** were recognized and accepted, albeit at a lower rate compared the enzymatically derived counterpart.^[116]^ A clear preference for Arg(42)‐ADP‐ribosylated ubiquitin was observed for the tested Legionella effectors.

**Scheme 10:**
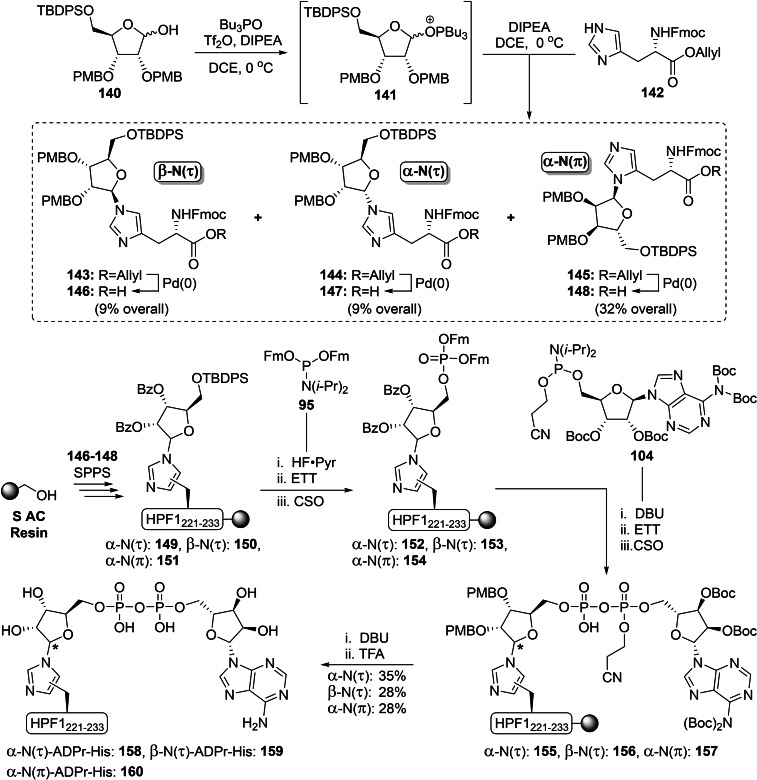
A synthetic approach towards three distinct ADP‐ribosylated histidine isomers where the key building blocks **146**–**148** were obtained via a base‐assisted Mukaiyama‐like glycosylation reaction with histidine. HPF1(221–233): T‐F‐Pra‐G‐A‐G‐L‐V‐V‐P‐V‐D‐K. Boc=*tert*‐butyloxycarbonyl, Bz=benzoyl, CSO=(1S)‐(+)‐(10‐camphorsulfonyl)‐oxaziridine, DBU=1,8‐diazabicyclo[5.4.0]undec‐7‐ene, ETT=5‐ethyl‐thio‐tetrazole, Fm=9‐fluorenylmethyl, Fmoc=fluorenylmethoxycarbonyl, *i*‐Pr=*iso*‐propyl, PMB=*para*‐methoxybenzyl, Pyr=pyridine, TBDPS=*tert*‐butylchlorodiphenylsilane, Tf=trifluoromethanesulfonyl and TFA=trifluoroacetic acid.

Although the imidate glycosylation strategy has facilitated the α‐selective ribosylation of a wide variety of amino acid functionalities,^[135]^ it proved to be incompatible with the basic imidazole functionality of histidine.^[150]^ No product formation was observed during the attempted acid‐catalyzed glycosylation reactions with TMSOTf, used either in catalytic or stoichiometric amounts, and silylation of the imidazole prior to glycosylation also was ineffective. Therefore glycosylation methodology was used that operates under basic conditions and we were drawn to a methodology described by Mukaiyama and co‐workers (Scheme 10).[^151^, ^152^] A bisphosphonium salt was generated from tributylphosphineoxide and triflic anhydride, which in turn reacted with the anomeric alcohol of a suitably protected ribofuranose (e. g. **140**) under aegis of the non‐nucleophilic base DIPEA to form the reactive phosphonium ribofuranoside intermediate **141**. This was slowly added to an excess of histidine derivative **142** in the presence of additional DIPEA to minimize over‐ribosylation. Under these optimized reaction conditions, three distinct mono‐ribosylated products were obtained in a 3 : 1 : 1 ratio and these were isolated after deprotection of the allyl ester by tetrakis(triphenylphospine)palladium and DMBA. Extensive NMR analysis revealed that the major product of the reaction (32 % overall) was the α‐configured N(π)‐regioisomer (**145**), while the minor products were obtained in identical amounts (9 % each) and were identified as the α‐N(τ) (**144**) and β‐N(τ) (**143**) isomers.

All three building blocks were successfully incorporated in an oligopeptide originating from HPF1 (**149**–**151**) using Fmoc‐based SPPS chemistry on an S AC resin, followed by the on‐resin construction of the ADP‐ribosyl modification. HF‐pyridine was used as a mild fluorine source to deprotect the TBDPS group without affecting ester linkages (including the *C*‐terminal bond to the resin). The primary alcohol was phosphitylated with Fm‐protected amidite **85** upon activation with ETT and subsequently oxidized with CSO to yield protected phosphorylribosides **152**–**154**. Then, deprotection of the phosphate group with DBU was followed by the two‐step P(V)‐P(III) pyrophosphate construction^[138]^ with Boc‐protected adenosine amidite **104** ^[142]^ to provide the partially protected ADPr peptides **155**–**157**. Finally, elimination of the 2‐cyanoethyl group was performed with DBU followed by the global deprotection and simultaneous cleavage from the resin under acidic conditions. Thanks to the high chemical stability of the constructs, all three peptides α‐N(τ) (**158**), β‐N(τ) (**159**) and α‐N(π) (**160**) ADP‐ribosylated on histidine could be obtained in high purity by reverse‐phase HPLC in yields of 28–35 %. The chemical (acid, base and nucleophilic reagents) and enzymatic stability of the His‐ADPr modification was investigated. All three peptides **158**–**160** were found to be surprisingly stable under all tested conditions, and none of the known human (ADP‐ribosyl)hydrolases were able to cleave the glycosidic linkage.^[150]^ The latter finding suggests the existence of a yet unknown enzyme with the capability to reverse the modification from histidine.

### 3.3 Chemical Synthesis of Artificial ADP‐Ribose Linkages

In addition to peptides featuring the native ADP‐ribosylated amino acids, several strategies have been developed for the preparation of peptide conjugates that are site‐specifically ADP‐ribosylated via non‐natural linkages. These strategies have been developed to facilitate the synthetic accessibility of ADP‐ribosylated peptides and to modulate their properties such as the stability of the protein‐ribose linkage.

#### 3.3.1 Convergent Syntheses of ADPr Mimetics: Oxime Ligation and Click Chemistry

**Scheme 11:**
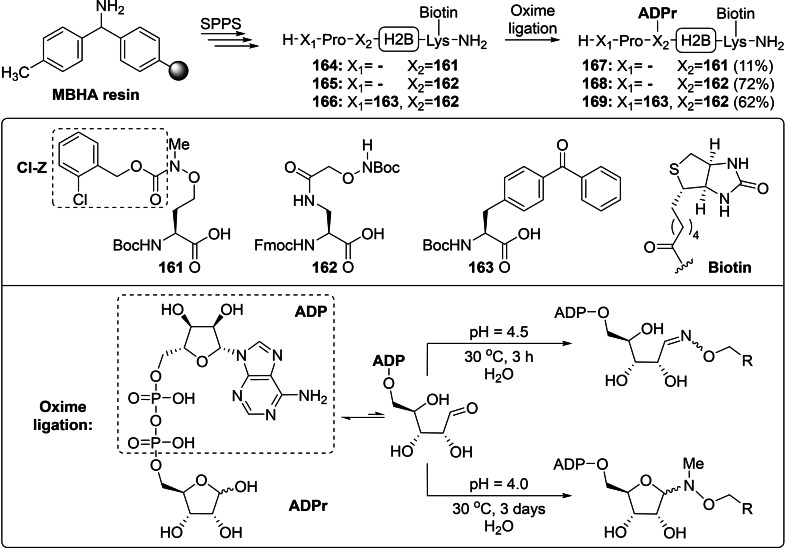
Site‐specific conjugation of ADP‐ribose to aminooxy or *N*‐methyl aminooxy functionalized histone H2B tail fragments by Moyle and Muir. Boc‐SPPS was applied for the assembly of **164** and **165**, while Fmoc‐based conditions were used for **166**. H2B_3–19_ sequence=PAK SAPAP KKGSK KAVT. Boc=*tert*‐butyloxycarbonyl, Fmoc=fluorenylmethoxycarbonyl and SPPS=solid‐phase peptide synthesis.

Over a decade ago, Moyle and Muir reported a methodology based on the chemoselective conjugation of free ADPr to aminooxy‐containing amino acids that were incorporated using building blocks **161** and **162** (Scheme 11) to deliver stable analogues for ester‐linked ADPr‐peptides.^[8]^ Adducts derived from secondary alkoxyamines and reducing carbohydrates exclusively adopt a ring‐closed form,^[153]^ as opposed to aminooxy groups that mainly generate a ring‐opened system upon reaction with linear chain carbohydrate aldehydes.^[154]^ The *N*‐methyl aminooxy building block **161** was derived from L‐homoserine according to known literature procedures^[155]^ and subsequently appended to the *N*‐terminal fragment (3–19) of histone H2B, functionalized with a biotin moiety for streptavidin pull‐down purposes, through Fmoc‐based SPPS on a 4‐methylbenzhydrylamine (MBHA) resin using HBTU/DIPEA. Ligation of the resulting peptide **164** to ADPr was performed in a sodium acetated buffered system (0.5 M, pH=4.0) to render all lysine and arginine residues unreactive. Despite a large excess of ADPr, the reaction did not reach completion with 40 % conversion after 3 whole days. The use of aniline (0.1–100 mM) as nucleophilic catalyst^[156]^ only promoted non‐specific reactions and lowering sodium acetate concentrations slowed down the reaction rate and reduced product yields. As a result, after preparative HPLC an inadequate amount of ADPr conjugate **167** was obtained to allow for detailed biochemical evaluation. H2B peptide **165** was assembled with commercially available *N*‐Boc‐aminooxyacetyl **162** via the same procedure, while Boc‐chemistry was used to incorporate **162** alongside benzophenone **163** in bifunctional peptide **166**. Addition of the photo‐induced cross‐linker would enable covalent attachment of the probe to low affinity ADPr‐binding proteins and possibly aid in the identification of new interaction partners. The conjugation of ADPr to peptides **165** and **166** was completed in less than 1 h and provided, according to spectroscopic analysis, a mixture of E/Z‐configured oxime products **168** and **169** in a 72 % and 62 % yield respectively. This demonstrated that oxime ligation under acidic conditions can be applied to efficiently produce ADP‐ribosylated conjugates. Constructs **167**–**169** were shown to be suitable substrates for PARP1‐mediated ADPr chain elongation and tightly interacted with the *macro*H2 A1.1 ADPr‐binder, while illustrating superior overall stability compared to the ester‐linked counterpart.^[8]^ Additionally, the application of benzophenone‐modified peptide **169** in the enrichment of ADPr‐binding proteins has been demonstrated.^[8]^

**Scheme 12:**
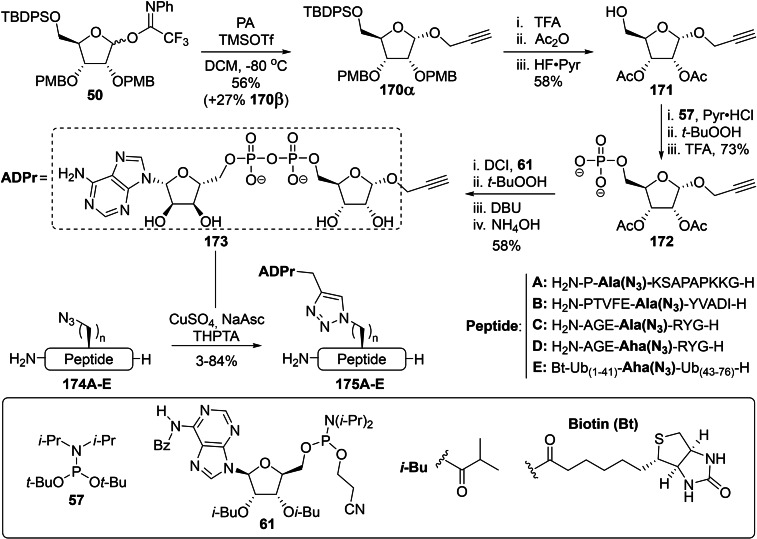
Preparation of triazole‐linked ADPr peptides and full‐length ubiquitin via a CuAAC between alkyne modified ADPr **173** and the target sequences **174**, equipped with an azide click handle. β‐azidoalanine (n=1) and β‐azidohomoalanine (n=2) are abbreviated as A(N_3_) and Aha(N_3_) respectively. Ac=acetyl, DBU=1,8‐diazabicyclo[5.4.0]undec‐7‐ene, DCI=dicyanoimidazole, *i‐*Pr=*iso‐*propyl, NaAsc=sodium ascorbate, PA=propargyl alcohol, Ph=phenyl, PMB=*para*‐methoxybenzyl, Pyr=pyridine, *t‐*Bu=*tert‐*butyl, TBDPS=*tert*‐butylchlorodiphenylsilane, TFA=trifluoroacetic acid, THPTA=tris(hydroxypropyl‐triazolylmethylamine) and Ub=ubiquitin.

Another general strategy for the post‐synthesis introduction of a single ADPr moiety on peptides of interest was developed by Liu *et al*., exploiting the versatile Cu(I)‐catalyzed azide‐alkyne cycloaddition (CuAAC) where alkyne‐ADPr analogue **173** was prepared and conjugated to an azide‐modified peptide sequence of interest (Scheme 12).^[157]^ The synthesis of alkyne ADPr **173** was initiated with the condensation of propargyl alcohol with acetimidate donor **50**,^[134]^ which provided a mixture of anomers (α/β=71 : 29) that were separable by silica gel column chromatography. The relevant α‐configured anomer **170** was subsequently converted into acetylated ribofuranoside **171** via a series of protecting group manipulations in an overall yield of 58 %. A two‐step phosphorylation procedure with *t*‐Bu protected amidite **61** was followed by immediate removal of the *t*‐Bu protecting groups using TFA to furnish phosphomonoester **172**. Pyrophosphate construction was achieved via a DCI‐promoted P(V)‐P(III) coupling with suitably protected adenosine phosphoramidite **61** and subsequent oxidation of the resulting intermediate with *t*‐BuOOH. Removal of the cyanoethyl group on the pyrophosphate with DBU and global deprotection of the ester‐linked functionalities in NH_4_OH finally provided propargyl‐ADPr **173**. To assess the viability of the projected cycloaddition, short peptide fragments **174A**–**D** were synthesized using standard Fmoc‐based SPPS conditions, incorporating the noncanonical β‐azidoalanine or β‐azidohomoalanine at the site of modification. The reactive Cu(I)‐catalyst, which was prepared *in situ* from Cu(II)SO_4_ with an excess of the reducing agent sodium ascorbate, was stabilized by tridentate ligand THPTA and added to the alkyne/azide mixture in tris(hydroxymethyl)‐aminomethane‐buffered saline (pH=7.6). All four click reactions proceeded efficiently, with complete conversion in less than 1 hour, and furnished the desired ADP‐ribosylated conjugates **175A**–**D** after HPLC purification. To investigate the versatility of the established protocol for the MARylation of oligopeptides, full‐length ubiquitin modified with a β‐azidohomoalanine **174E** was synthetically derived.^[158]^ The CuAAC reaction was successfully performed under identical conditions as described for the peptide fragments. The excess of propargyl ADPr **173** and click reagents were removed by dialysis, after which size exclusion chromatography gave the desired ubiquitin conjugate **175E** in high purity in 84 % yield. The triazolyl‐linked ADPr ubiquitin **175E** was efficiently visualized by an ADPr‐antibody on a Western blot, which allowed the monitoring of the auto‐ubiquitination of *Legionella* effector SdeA. Significant auto‐ubiquitination was observed with **115E**, albeit somewhat slower than for its naturally derived counterpart.^[157]^

**Scheme 13:**
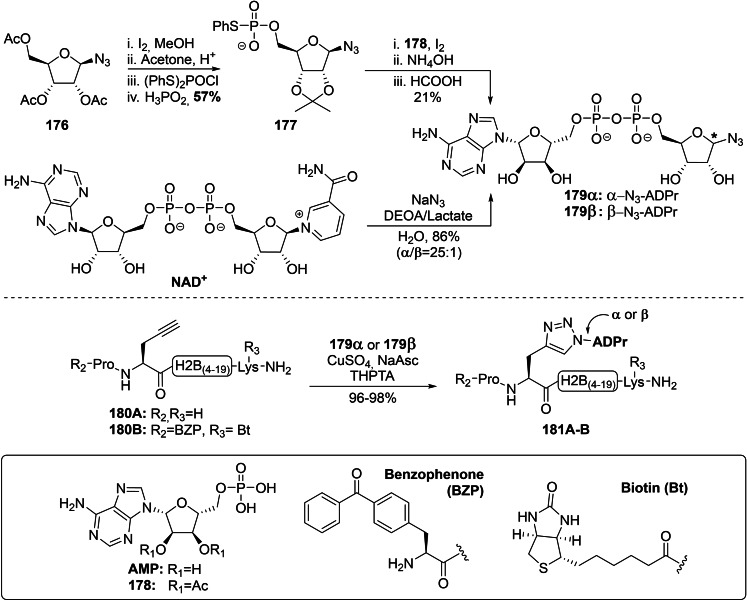
Modular synthesis of ADP‐ribosylated histone H2B fragments via a CuAAC between ADPr‐N_3_ analogues and the target peptide with a propargylglycine at the modification site by Li and Zhang and coworkers. H2B_4–19_ sequence=P‐A‐K‐S‐A‐P‐A‐P‐K‐K‐G‐S‐K‐K‐A‐V‐T. Ac=acetyl, DEOA=diethanol‐amine, NaAsc=sodium ascorbate, Ph=phenyl, and THPTA=tris(hydroxypropyltriazolylmethylamine).

Li *et al*. were the first ones to exploit the CuAAC for the preparation of artificial ADP‐ribosylated oligopeptides and opted, in contrast to the alkyne‐modified ADPr analogues of Liu (Scheme 12), for azido‐ADPr analogues **179** as key components for the late‐stage modification strategy (Scheme 13). Initial efforts were focused on the development of a synthetic route towards β‐N_3_‐ADPr **179β** from its azido‐ribofuranoside precursor **176**.[^149^, ^159^] Deacetylation with iodine in methanol and subsequent introduction of the isopropylidene group under acidic conditions was followed by efficient phosphorylation of the 5‐OH group with (PhS)_2_POCl in the presence of tetrazole as nucleophilic catalyst. Partial deprotection of the dithiophosphate intermediate with hypophosphorous acid in pyridine furnished phenylthiophosphate **177**, which was then subjected to the iodine‐promoted condensation reaction with 2‐,3‐acetylated AMP **178**.^[160]^ Subsequent treatment with NH_4_OH and formic acid facilitated removal of the remaining acetyl and acetonide protecting groups to furnish azido ADPr **179β** in 12 % overall yield over 8 steps after preparative HPLC.

Several years later, Zhu *et al*. developed an α‐selective ribosylation reaction that enabled the direct conversion of NAD^+^ into the biologically more relevant N_3_‐ADPr anomer **179α** using an ionic liquid/water system.^[117]^ The right mixture of cations and anions in ionic liquids have previously been shown to facilitate otherwise impossible chemical transformations through synergistic catalytic effects such as the stabilization of reactive intermediates via noncovalent interactions.[^161^, ^162^] After optimization of the water content and reaction temperature, an elaborate screening of 54 different ionic liquid systems was performed in which the combination of diethanolamine/lactate was found to be superior to achieve optimal α‐selectivity. Increasing the scale of the reaction (0.5 gram) did not reduce the stereoselectivity nor the reaction yield and provided α‐N_3_‐ADPr **179α** in an excellent yield of 86 % (α/β=25 : 1) after a desalination column to remove excess sodium azide. The alkyne‐modified amino‐terminus of histone H2B **180A** as well as its bifunctional derivative **180B**, decorated with a biotin and photo‐cross‐linker, were selected as model peptides and these were obtained from commercial sources. During optimization of the click reaction conditions with phenylacetylene as test substrate, it was demonstrated that the *in situ* derived Cu(I)‐species was most effectively stabilized by the tridentate ligand tris(benzyltriazolylmethyl)amine (THPTA)^[163]^ in the employed water/ethanol/*tert*‐butanol (2 : 3 : 5) solvent system, outperforming the tris(benzyltriazolyl‐methyl)amine (TBTA)^[164]^ and (bis(benzyltriazolylmethyl)amino)‐*N*‐methylacetamide BBTA^[165]^ ligands. Application of the optimized CuAAC conditions efficiently conjugated H2B peptides **181** to α‐ or β‐configured N_3_‐ADPr analogues and provided a total of four ADP‐ribosylated peptide conjugates (**α‐181A**, **α‐181B**, and **β‐181A** and **β‐181B**) in near quantitative yields after preparative HPLC purification. Similar to the proof‐of‐concept experiments performed by Moyle and Muir,^[8]^ Li *et al*. demonstrated the binding of triazolyl‐linked isosteres **181** to macrodomain mH2 A1.1, PARP1‐mediated chain elongation on peptides **181** as well as their potential in the enrichment of ADPr‐binders from HeLa lysate.^[117]^

**Scheme 14:**
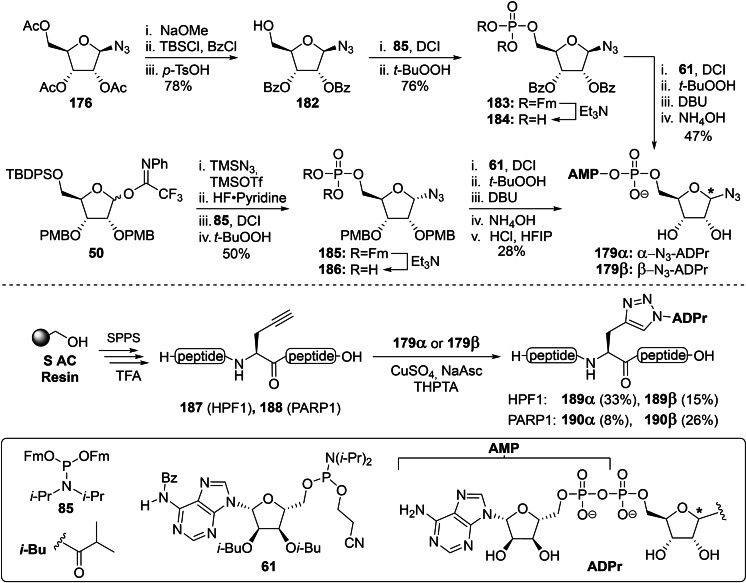
An alternative route towards a‐ and b‐configured azido‐ADPr analogues and their implementation via a late‐stage Cu(I)‐catalyzed conjugation in peptide fragments HPF1(221–233): T‐F‐**Pra**‐G‐A‐G‐L‐V−V‐P‐V‐D‐K and PARP1(529–553): G‐G‐A‐A‐V‐D‐P‐D‐S‐G‐L‐E‐Pra‐S−A. The α/β assignment involves the anomeric centre highlighted by *. Ac=acetyl, Bz=benzoyl, DBU=1,8‐Diazabicyclo(5.4.0)undec‐7‐ene, Fm=9‐fluorenylmethyl, *i‐*Bu=*i*so‐butyl, *i‐*Pr=*iso*‐propyl, NaAsc=sodium ascorbate, PMB=*para*‐methoxybenzyl, Pra=propargylglycine, *p*‐TsOH=*para*‐toluenesulfonic acid, SPPS=solid‐phase peptide synthesis, TBDPS=*tert*‐butylchlorodiphenylsilane, TBS=*tert*‐butylsilyl, TMSOTf=trimethylsilyl trifluoromethanesulfonate, and THPTA=tris(hydroxypropyltriazolylmethylamine).

Shortly after the ionic liquid strategy of Zhu *et al*., Minnee *et al*. described a total synthesis of azido‐ADPr derivatives **179α** and **179β** (Scheme 14)^[166]^ to investigate their potential as isosteres for the recently observed ADP‐ribosylated histidine sites.^[72]^ For the preparation of **759β**, the acetyl groups of β‐azido‐ribofuranoside **176** ^[149]^ were removed under basic conditions to permit the installation of orthogonal protecting groups. To this end, a TBS group was selectively introduced on the primary alcohol, which was directly followed by the installation of benzoyl moieties on the 2‐ and 3‐OH via a two‐step one‐pot procedure in pyridine. Removal of the silyl ether under acidic conditions provided the primary alcohol **182**, which could be efficiently phosphitylated with amidite **85** in the presence of DCI. The P(III)‐intermediate was immediately oxidized with *t*‐BuOOH to give 1‐β‐azido‐5‐phosphorylribofuranoside **183** in a 76 % yield. The phosphate was liberated from its Fm‐protecting groups by treatment with triethylamine. Using the well‐established P(III)‐P(V) coupling method,^[138]^ freed phosphate **184** was conjugated to adenosine phosphoramidite **61** ^[167]^ upon activation with DCI and subsequently oxidized with *t*‐BuOOH. The cyanoethyl group was eliminated from the pyrophosphate with DBU followed by a global deprotection in aqueous ammonia to yield β‐N_3_‐ADPr **179β** in a 47 % yield over 5 steps, after size exclusion chromatography.

In contrast to its β‐configured counterpart, a non‐participating group at the 2‐OH position is required for the assembly of α‐configured azido‐5‐phosphorylribofuranose **185**. *N*‐Phenyl trifluoroacetimidate donor **50** designed by Kistemaker *et al*.^[135]^ demonstrated its worth once more and reacted in excellent stereoselectivity (α/β=14 : 1) with TMSN_3_ at −60 °C upon activation of the donor with TMSOTf. Removal of the PMB groups under either acidic or oxidative conditions proved difficult at this point and was therefore postponed to a later stage. Instead, the TBDPS groups was eliminated with HF‐pyridine as fluorine source and the freed primary alcohol was phosphorylated using identical conditions as described for the β‐azide to yield α‐phosphorylribofuranoside **185** in 50 % yield over 4 steps. The same reaction sequence could be applied to generate the 2’’,3’’‐*O*‐PMB‐protected α‐N_3_‐ADPr precursor, which was purified using reversed‐phase HPLC. Careful deprotection of the PMB ethers was realized at this stage using a catalytic amount of HCl in HFIP^[136]^ to yield azido‐ADPr **179α**.

The selected oligopeptides, originating from HPF1 (**187**) and PARP1 (**188**),^[72]^ were assembled on an S AC resin via the standard Fmoc‐based SPPS strategy with DIC/oxyma as coupling reagents, where the original histidine residue was substituted for propargylglycine. Finally, the alkyne‐modified peptides were conjugated to β‐ or α‐N_3_‐ADPr (1.5‐fold molar excess) through a CuAAC. To ensure consistent and efficient cycloadditions, the active Cu(I)‐catalyst was generated *in situ* before every reaction by mixing an aqueous solution of Cu(II)SO_4_ to sodium ascorbate and immediately adding the tridentate ligand THPTA. Upon completion of the CuAAC reaction, a tandem purification strategy involving size exclusion chromatography followed by reversed‐phase HPLC allowed isolation of the desired products, but in moderate yields only. Direct preparative HPLC proved to be more efficient and provided the desired triazolyl‐linked ADPr constructs **189**–**190** in high purity with yields up to 33 %. The promiscuous nature of ARH3 was once more highlighted in a screening of triazolyl‐ADPr peptides **189**–**190** against a library of human (ADP‐ribosyl)hydrolases, where a slow but steady conversion of **189α** was observed.^[166]^ Surprisingly, PARP1 derived fragment **190α** remained unaffected, highlighting the sequence dependence of the ARH3 hydrolytic activity.^[166]^

#### 3.3.2 Step‐Wise On‐Resin Syntheses of ADPr Mimetics

**Scheme 15:**
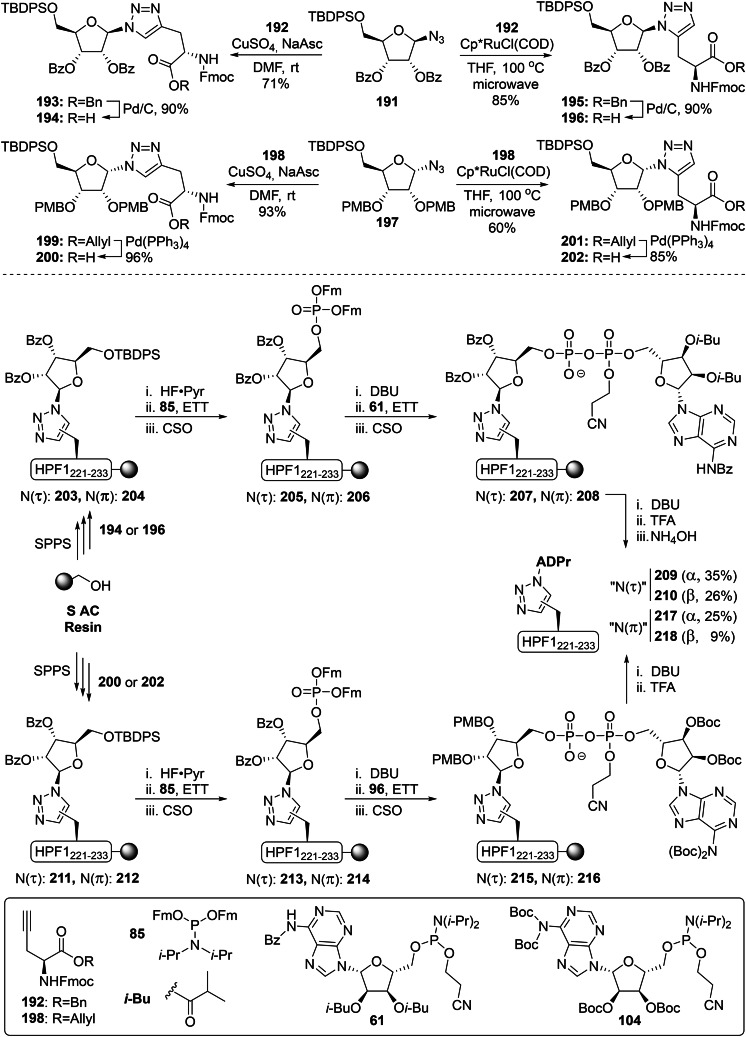
An SPPS‐based approach towards a complete set of 1,4‐ and 1,5‐triazolyl linked ADP‐ribosylated peptide fragments by exploiting both Cu(I)‐ and Ru(II)‐catalyzed click chemistry. HPF1(221–233): T‐F‐**H**‐G‐A‐G‐L‐V‐V‐P‐V‐D‐K, where the site of modification is depicted bold. Bz=benzoyl, Boc=*tert*‐butyloxycarbonyl, Bn=benzyl, Cp*=cyclopentadienyl, CSO=(1S)‐(+)‐(10‐camphorsulfonyl)‐oxaziridine, DBU=1,8‐Diazabicyclo(5.4.0)undec‐7‐ene, ETT=5‐(Ethylthio)‐1H‐tetrazole, Fm=9‐fluorenylmethyl, Fmoc=fluorenylmethoxycarbonyl, *i‐*Bu=*iso‐*butyl, *i‐*Pr=*iso‐*propyl, PMB=*para*‐methoxybenzyl, Pyr=pyridine, TBDPS=*tert*‐butylchlorodiphenylsilane, and TFA=trifluoroacetic acid.

In order to generate isosteres for both possible regioisomers of ADP‐ribosylated histidine, Minnee *et al*. aimed to expand the collection of 1,4‐triazolyl‐linked ADPr conjugates with their respective 1,5‐triazole equivalents by exploiting the less common Ru(II)‐catalyzed azide‐alkyne cyclcoaddition (RuAAC, Scheme 15).^[168]^ Due to the incompatiblity of the RuAAC reaction conditions with a late‐stage coupling, another strategy had to be developed. Instead, β‐azido‐ribofuranoside **191** and α‐configured analogue **177** were added to an orthogonally protected propargylglycine derivative (**192** and **198** respectively). The generation of the 1,5‐regioisomers under catalysis of chloro(pentamethylcyclo‐pentadienyl)(cyclooctadiene)ruthenium(II) (Cp*RuCl(COD)) in THF required an elevated temperature of 100 °C for efficient conversion, but effectively provided triazoles **175** and **201**. The regioisomeric compounds **193** and **199** were generated in parallel using a Cu(I)‐mediated cycloaddition. Hydrogenolysis of the benzyl esters with Pd/C or deallylation with tetrakis(triphenylphosphine)‐palladium in the presence of DMBA as nucleophilic scavenger finally provided a the set of four key intermediates (**194**, **196**, **200** and **202**) compatible with SPPS chemistry.

A peptide derived from HPF1 was assembled with the newly obtained 1,4‐ and 1,5‐ribosylated building blocks at the position of histidine using DIC/oxyma‐mediated couplings on an S AC resin. The fully protected intermediates **203**–**204** (β) and **211**–**212** (α) were initially subjected to TBAF to remove the silyl ether.^[115]^ However, LC–MS analysis indicated that the reaction was troubled by partial deprotection of the 2‐/3‐*O*‐benzoyl moieties, thereby suggesting that the *C*‐terminal ester linkage to the resin is also at risk which could lead to significant loss of material. A milder alternative for this deprotection was found in HF‐pyridine, which left the ester functionalities unscathed. The primary alcohol was phosphorylated via a two‐step procedure with Fm‐protected amidite **85** under the aegis of ETT, and subsequent CSO‐ oxidation to provide the protected phosphates **205**–**206** (β) and **213**–**214** (α). Liberation of the phosphate with DBU then furnished the P(V)‐precursor.^[138]^ The β‐isomers were coupled to adenosine phosphoramidite **61**, while the α‐configured analogues were coupled to an adenosine phosphoramidite equipped with acid‐labile protecting groups (**104**)^[142]^ to simplify the final stage of the on‐resin synthesis. The immobilized ADPr conjugates **207**–**208** (β) and **215**–**216** (α) were first treated with DBU to eliminate the 2‐cyanoethyl moiety and then subjected to 50 v/v% TFA/DCM to ensure cleavage from the Tentagel resin and simultaneous removal of the *tert*‐butyl carbamate (Boc) groups and PMB ethers in case of α‐isomers **217**–**218**. To facilitate deprotection of all the remaining base‐sensitive groups from the β‐configured analogues, they were stirred in aqueous ammonia. HPLC purification (using an NH_4_OAc based eluent system) furnished the four triazolyl‐linked ADPr isomers **209**–**210** as ammonium salts in yields up to 35 %. The triazolyl‐linked isosteres **209**–**210** remained intact in the presence of hydroxylamine or under strong acidic conditions (TFA), but were found to be prone to NaOH catalyzed isomerization. An assay with ARH3 demonstrated that this hydrolase was much more efficient in removing the ADPr modification from the N(π)‐position than from the N(τ)‐position.^[168]^ However, this observation is devoid of biological relevance since ADPr−N(π)‐His **157** (Scheme 10) is not processed by ARH3 or any other known human ADPr‐hydrolase.^[168]^

**Scheme 16:**
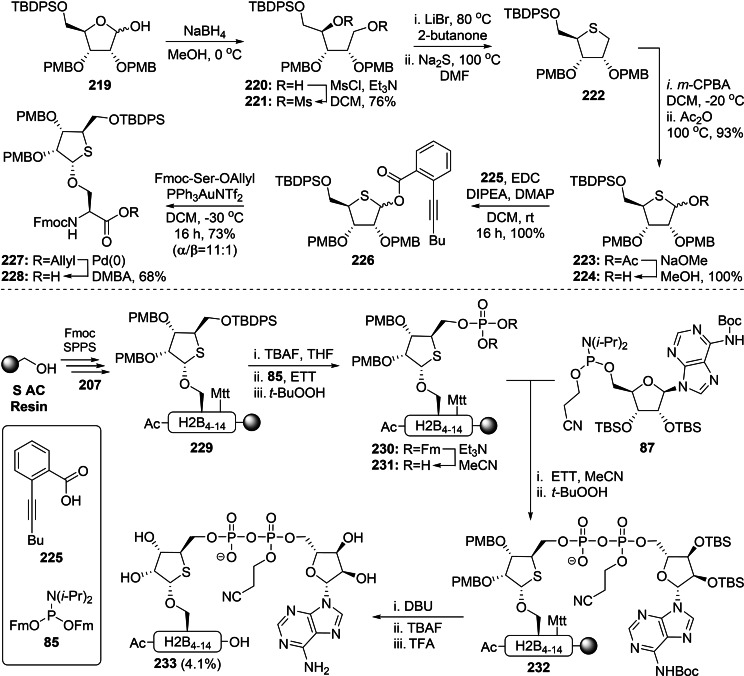
The use of an Au(II)‐mediated glycosylation reaction in the synthesis of ‐configured thio‐furanoside on serine and its ensuing incorporation in the *N*‐terminus fragment of H2B(4–14): P‐A‐K‐**S**‐A‐P‐A‐P‐K‐K‐G, where the site of the modification in highlighted bold. Boc=*tert*‐butyloxycarbonyl, Bu=butyl, DBU=1,8‐Diazabicyclo(5.4.0)undec‐7‐ene, DMBA=1,3‐dimethylbarbituric acid, ETT=5‐(Ethylthio)‐1H‐tetrazole, Fm=9‐ fluorenylmethyl, Fmoc=fluorenylmethoxycarbonyl, *i‐*Pr=*iso*Pr, m‐CPBA=meta‐Chloroperoxybenzoic acid, MsCl=methanesulfonyl chloride, Mtt=4‐methyltrityl, PMB=*para*‐methoxylbenzyl, TBAF=tetrabutylammonium fluoride, TBDPS=*tert*‐butylchlorodiphenylsilane, TBS=*tert*‐butylsilyl, Tf=triflyl and TFA=trifluoroacetic acid.

In search for a stabilized mimetic of ADP‐ribosylated serine, which is sensitive to enzymatic cleavage,^[9]^ Madern *et al*. reasoned that the substitution ring oxygen by a sulfur atom could improve resistance to acidic and enzymatic degradation.[^169^, ^170^] The synthesis of 4‐thio ribosyl serine building block **228** (Scheme 16) was initiated from orthogonally protected lactol **219**[^134^, ^171^] by reducing the aldehyde functionality in the open‐chain form of ribose with sodium borohydride. According to a strategy developed by Minakawa *et al*.,[^172^, ^173^] the resulting diol **220** was then mesylated (**221**) and subjected to LiBr to generate the 1,4‐dibromide. This inversion is necessary to retain the original stereochemistry at the C‐4 position upon substitution of the bromides and the accompanying ring closure by Na_2_S, which yielded 1‐deoxy‐thioriboside **222**. Oxidation of the thioether was realized by *meta*‐chloroperoxybenzoic acid at −40 °C, after which the intermediate sulfoxide was converted into 1‐acetyl‐thioribofuranoside **223** via a Pummerer rearrangement. Removal of the anomeric acetyl group with NaOMe then provided thioribofuranoside **224** in quantitative yield. Unfortunately, glycosylation attempts with the *N*‐phenyl trifluoroacetimidate derivative^[135]^ of **224** under the (Lewis)‐acidic conditions (TMSOTf, TBSOTf and HClO_4_‐SiO_2_) did not lead to the target serine derivative, but predominantly resulted in a PMB transfer from the donor to the hydroxy group of serine. A milder, gold‐mediated strategy was therefore explored,^[174]^ which required the preparation of alkynylbenzoate donor **226** via an EDC coupling with *O*‐hexynylbenzoic acid **225**. The coupling of the Fmoc‐Ser‐OAllyl acceptor to donor **225** was succesfully facilitated by a catalytic amount of PPh_3_AuNTf_2_ in a 73 % yield with satisfying α‐selectivity (α/β=11 : 1). Finally, the allyl ester was deprotected using tetrakis(triphenylphosphine)palladium and DMBA to furnish the SPPS‐compatible intermediate **228**.

The *N*‐terminus of histone H2B(4–14) was assembled on S AC resin using HCTU/DIPEA‐mediated peptide couplings, where serine was exchanged for the newly prepared thio‐ribosylated building block **228**. To install the pyrophosphate, the primary silyl ether had to be removed and in a direct comparison with HF‐pyridine and HF‐triethylamine, TBAF was found to be the superior desilylation reagent in terms of reaction rate and quality of the intermediate product as determined by LC–MS analysis. The freed primary alcohol was phosphitylated with Fm‐protected amidite **85** upon activation with ETT. Test reactions demonstrated that CSO would oxidize both the phosphite intermediate and the thioether. Fortunately, the milder oxidizing reagent *t*‐BuOOH could be used to selectively oxidize the phosphite group to furnish **230**. After removal of the Fm‐protecting groups with DBU, the liberated phosphate (**231**) was coupled to adenosine phosphoramidite **87** using P(III)‐P(V) chemistry^[138]^ and subsequently oxidized with *t*‐BuOOH to prevent formation of the sulfoxide side product. The final deprotection reaction sequence of **232** was intitiated with DBU to eliminate the 2‐cyanoethyl group, followed by a TBAF treatment to deprotect the 2’‐ and 3’‐*O*‐silyl ethers and finally 10 % TFA to remove the remaining acid‐labile protecting groups and concomitant cleavage from the resin. As a result, thio‐ADP‐ribosylated peptide **233** was obtained in 4.1 % overall yield, after purification by reversed‐phase HPLC. The *O*‐glycosidic linkage in Ser‐thio‐ADPr **233** was found to be much more stable towards ARH3 with a mere 3 % hydrolysis compared to the native Ser‐ADPr.^[170]^ This finding indicates a strong stabilizing effect of the thio‐sugar on the glycosidic bond.

## 4 Summary and Outlook

Overall, the collective of chemoenzymatic and chemical methodologies described here has covered a significant portion of the different MARylation possibilities during the last three decades. Despite their limitation to a relatively small subset of residues, enzymatic approaches have proven their worth in the scalable and highly efficient modification of serine, tyrosine and acidic amino acid residues. More importantly, all these methods, both chemical and chemoenzymatic, have been made compatible with downstream applications such as NCL, which has enabled the construction of various full‐length histones carrying a site‐specific ADPr modification. An increased understanding of the mechanisms determining substrate selectivity of (ADP‐ribosyl) transferases and, more specifically, the discovery of additional co‐factors like HPF1 will potentially broaden the scope of accessible ADP‐ribosylation sites in the future. It is noteworthy that enzyme‐assisted approaches are, at present, indispensable because the preparation of PARylated oligopeptides^[123]^ has not been attainable so far using fully synthetic means.

Significant advancements have been made to generate close analogues of ADPr proteins and peptides. A clear advantage of the convergent syntheses where ADPr moieties are introduced through CuAAC, RuAAC or oxime ligation, is the ease at which a library of ADPr peptides can be generated from relatively few, and sometimes commercially available, precursors. However, these artificially linked ADP‐conjugates require thorough evaluation of their properties prior to their use in biological settings and their results always have to be interpreted with caution. While the on‐resin ADPr synthesis approach to generate nature‐identical ADPr peptides was initially highly laborious and troubled by the formation of contaminations that were difficult to remove, it has matured in a stepwise manner into an effective and versatile means to generate well‐defined MARylated peptides with the successful optimization of phosphoramidite chemistry and carefully devised protecting group strategies. The SPPS‐based methodologies have enabled the introduction of ADPr on a substantial fraction of the known ADP‐ribosyl acceptor sites and will likely be the basis for new strategies towards the modification of currently missing residues such as lysine as well as the installation of novel ADPr mimetics. Finally, the combination of the on‐resin construction of MARylated peptides with the chain elongation capability of PARP1 could potentially cover the entire poly‐ADP‐ribosylome.

## Conflict of Interests

The authors declare no conflict of interest.

## Biographical Information

*Hugo Minnee is a PhD student at Leiden University under the supervision of Prof. Jeroen Codée and Dr. Dmitri Filippov. He received his B.Sc. in ‘Molecular Science and Technology (MST)’ from Leiden University and TU Delft in 2015 and his M.Sc. in Chemistry from Leiden University in 2018. His doctoral research is dedicated to the development of synthetic methodologies towards peptides ADP‐ribosylated on histidine and analogues thereof*.

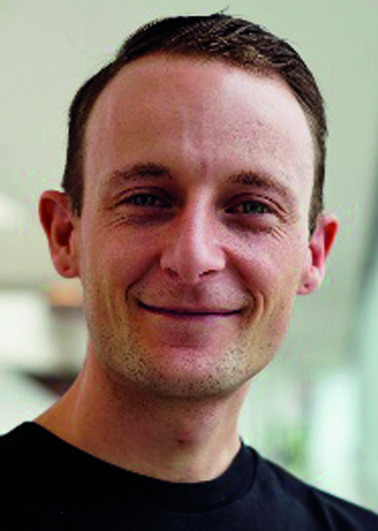

## Biographical Information

*Jeroen Codée obtained his PhD from Leiden University under the guidance of Prof. Jacques van Boom and Prof. Stan van Boeckel. After a post‐doctoral stay at the Eidgenössische Technische Hochschule (ETH) Zürich he returned to Leiden University, where he now is a Professor of Organic Chemistry in the bioorganic synthesis group. His research is focused on glycochemistry and chemical glycobiology spanning from unravelling the intricate details of the chemical glycosylation reaction, to the assembly of complex (bacterial) oligosaccharides and glycoconjugates and the development of molecular tools to study glycoprocessing enzymes and the development of (mechanism‐based) inhibitors*.

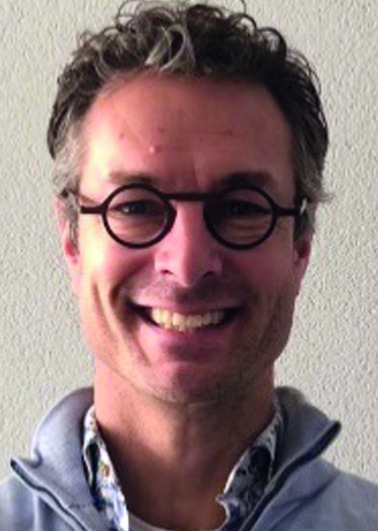

## Biographical Information

*Dmitri Filippov obtained his Ph.D. degree from Leiden University (1998) under the guidance of Jacques van Boom and Gijs van der Marel while he was investigating the application of phosphoramidite reagents to the synthesis of peptide–nucleic acid hybrid biopolymers. Dmitri has remained in Leiden ever since where he currently is an Associate Professor. His research interests include synthetic bioorganic chemistry with a focus on the chemistry of ADP‐ribosylated biomolecules and other hypermodified peptides and nucleic acids, both native and artificial*.